# Comparison of clinical outcomes between aggressive and non-aggressive intravenous hydration for acute pancreatitis: a systematic review and meta-analysis

**DOI:** 10.1186/s13054-023-04401-0

**Published:** 2023-03-22

**Authors:** Xiu-Wei Li, Chien-Ho Wang, Jhih-Wei Dai, Shu-Han Tsao, Po-Hsi Wang, Cheng-Chen Tai, Rong-Nan Chien, Shih-Chieh Shao, Edward Chia-Cheng Lai

**Affiliations:** 1 Division of Hepatogastroenterology, Department of Internal Medicine, Keelung Chang Gung Memorial Hospital, Keelung, Taiwan; 2 College of Medicine, Chang Gung University, Taoyuan, Taiwan; 3 Department of Emergency Medicine, Keelung Chang Gung Memorial Hospital, Keelung, Taiwan; 4 Department of Cardiology, Keelung Chang Gung Memorial Hospital, Keelung, Taiwan; 5 Department of Urology, Linkou Chang Gung Memorial Hospital, Taoyuan, Taiwan; 6 Department of Nephrology, Keelung Chang Gung Memorial Hospital, Keelung, Taiwan; 7 Medical Library, Department of Medical Education, Linkou Chang Gung Memorial Hospital, Taoyuan, Taiwan; 8 Division of Hepatogastroenterology, Department of Internal Medicine, Linkou Chang Gung Memorial Hospital, Taoyuan, Taiwan; 9 Department of Pharmacy, Keelung Chang Gung Memorial Hospital, Keelung, Taiwan; 10 Center for Evidence-Based Medicine, Keelung Chang Gung Memorial Hospital, Keelung, Taiwan; 11 School of Pharmacy, Institute of Clinical Pharmacy and Pharmaceutical Sciences, College of Medicine, National Cheng Kung University, Tainan, Taiwan

**Keywords:** Aggressive intravenous hydration, Acute pancreatitis, Mortality, Systematic review, Meta-analysis

## Abstract

**Background:**

Current practice guidelines for optimal infusion rates during early intravenous hydration in patients with acute pancreatitis (AP) remain inconsistent. This systematic review and meta-analysis aimed to compare treatment outcomes between aggressive and non-aggressive intravenous hydration in severe and non-severe AP.

**Methods:**

This study followed the Preferred Reporting Items for Systematic Reviews and Meta-Analyses guidelines. We systematically searched PubMed, Embase and Cochrane Library for randomized controlled trials (RCTs) on November 23, 2022, and hand-searched the reference lists of included RCTs, relevant review articles and clinical guidelines. We included RCTs that compared clinical outcomes from aggressive and non-aggressive intravenous hydration in AP. Meta-analysis was performed using a random-effects model for participants with severe AP and non-severe AP. Our primary outcome was all-cause mortality, and several secondary outcomes included fluid-related complications, clinical improvement and APACHE II scores within 48 h.

**Results:**

We included a total of 9 RCTs with 953 participants. The meta-analysis indicated that, compared to non-aggressive intravenous hydration, aggressive intravenous hydration significantly increased mortality risk in severe AP (pooled RR: 2.45, 95% CI: 1.37, 4.40), while the result in non-severe AP was inconclusive (pooled RR: 2.26, 95% CI: 0.54, 9.44). However, aggressive intravenous hydration significantly increased fluid-related complication risk in both severe (pooled RR: 2.22, 95% CI 1.36, 3.63) and non-severe AP (pooled RR: 3.25, 95% CI: 1.53, 6.93). The meta-analysis indicated worse APACHE II scores (pooled mean difference: 3.31, 95% CI: 1.79, 4.84) in severe AP, and no increased likelihood of clinical improvement (pooled RR:1.20, 95% CI: 0.63, 2.29) in non-severe AP. Sensitivity analyses including only RCTs with goal-directed fluid therapy after initial fluid resuscitation therapy yielded consistent results.

**Conclusions:**

Aggressive intravenous hydration increased the mortality risk in severe AP, and fluid-related complication risk in both severe and non-severe AP. More conservative intravenous fluid resuscitation protocols for AP are suggested.

**Graphical Abstract:**

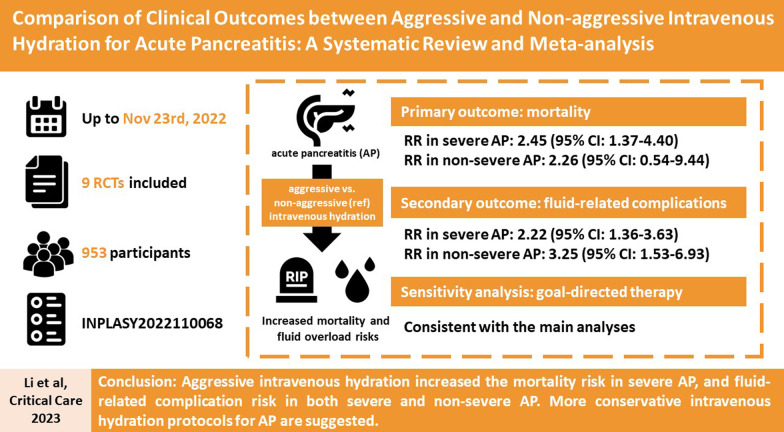

**Supplementary Information:**

The online version contains supplementary material available at 10.1186/s13054-023-04401-0.

## Introduction

Acute pancreatitis (AP) is one of the most frequent and critical gastrointestinal diseases resulting in hospital admissions worldwide [1–3]. The global incidence and mortality of acute pancreatitis is estimated at 34 cases per 100,000 person-years and 1.6 deaths per 100,000 person-years, respectively, and the incidence has been reported to be rising in recent years [4–6]. Appropriate initial management decisions for AP can significantly affect the disease course and hospitalization duration [5, 7–10].

According to a number of international guidelines, early fluid resuscitation with predominantly isotonic crystalloid (i.e., normal saline or Ringer’s lactate solution) is widely indicated for AP management to prevent hypovolemia and organ hypoperfusion, without waiting for hemodynamic worsening [7, 8, 11, 12]. However, the guidelines for the design of fluid resuscitation protocols remain inconsistent when it comes to the infusion rate [12–19]. For example, the American College of Gastroenterology (ACG) guidelines suggest that aggressive intravenous hydration (250–500 ml/hour) should be given to all patients with AP in the first 12–24 h unless cardiovascular and/or renal comorbidities exist [11]. For severe AP, the Italian Association for the Study of the Pancreas (AISP) suggests early aggressive hydration at 2 ml/kg/h, with an initial bolus of 20 ml/kg within 30–45 min in the first 24 h [20]. However, the guidelines of the American Gastroenterological Association (AGA) and experts from ACG’s Acute Pancreatitis Task Force suggest a goal-directed fluid therapy, but are unable to make specific recommendations on the optimal initial rate of fluid resuscitation in AP, due to the paucity of evidence [7, 21].

Three previous systematic review and meta-analysis studies, potentially with methodological flaws, yielded inconsistent findings on the effects of aggressive intravenous hydration in AP [22–24]. Gad MM et al. concluded that aggressive intravenous fluid therapy did not reduce mortality in AP, based on 9 included studies (3 randomized controlled trials (RCTs) and 6 cohort studies) [23], and Liao J et al. [22] concluded that aggressive hydration increases in-hospital mortality in AP by 1.66 times, based on 12 included studies (4 RCTs and 8 cohort studies). However, the certainty of evidence (CoE) from these reviews was compromised since the inclusion of observational studies with RCTs in meta-analyses frequently increases heterogeneity among the included studies and is therefore discouraged [25]. Another systematic review and meta-analysis from Di Martino M et al. [24] reported that high-rate fluid infusion increased mortality about threefold in AP, compared to moderate-rate fluid infusion, based on 4 RCTs. However, these previous meta-analyses did not separately analyze the severity of AP, so firm conclusions regarding specific AP populations cannot be drawn. Recently, interim findings from the WATERFALL trial indicated about threefold increased risks of fluid overload, and potentially threefold increased risks of mortality, in patients with non-severe AP receiving aggressive intravenous fluid resuscitation, compared to those receiving non-aggressive fluid resuscitation [26]. Since the heterogeneity, in terms of disease severity, of the population studied in previous RCTs has probably contributed to inconsistent findings, we conducted this systematic review and meta-analysis of RCTs, separately reporting and contrasting the benefits and harms of aggressive and non-aggressive intravenous hydration protocols for severe and non-severe AP.

## Methods

This systematic review and meta-analysis followed the Preferred Reporting Items for Systematic Reviews and Meta-Analyses (PRISMA) guidelines (Additional file 1: Appendix Table S1) [27], and the pre-defined study protocol has been published on the International Platform of Registered Systematic Review and Meta-analysis Protocols (INPLASY) with the registration number INPLASY2022110068.

### Search strategy

We searched PubMed, Embase and the Cochrane Library from database inception to November 23, 2022, with no limitation on language, to identify relevant RCTs. The search strategy was developed by an experienced evidence-based medicine researcher (SCS) in collaboration with a senior librarian (CCT) (Additional file 1: Appendix Table S2). Important keywords with MeSH terms included “acute pancreatitis”, “normal saline” and “Lactated Ringer’s solution.” To make our search more comprehensive, we also manually reviewed the reference lists of the included studies, previous review articles and published guidelines regarding AP.

### Inclusion criteria

After removing duplicate records from the different databases, two independent reviewers (XWL and CHW) selected the included studies based on the following PICOS criteria: (1) Participants: Adults with AP. The diagnosis of AP should be based on two of the following criteria developed by the Atlanta international symposium and revised Atlanta classification: a.) Abdominal pain consistent with AP (e.g., acute onset of persistent, severe, epigastric pain often radiating to the back); b.) Serum lipase or amylase activity at least three times greater than the upper limit of normal; or c.) Classic image findings from computed tomography, magnetic resonance imaging or abdominal ultrasonography [1, 28]. We defined the severity of AP based on the Atlanta international symposium and revised Atlanta classification [1, 28]. For example, mild AP is defined by the absence of organ failure and local or systemic complications, and moderately severe AP is defined by the presence of transient organ failure or local or systemic complications. We classified mild and moderately severe AP into the non-severe AP group, because severe AP, characterized by persistent organ failure, may pose a higher mortality risk [2]; (2) Interventions: aggressive intravenous fluid resuscitation, defined as a.) Fluid administration (predominantly normal saline or lactated Ringer’s solution) at a rate greater than 10 ml/kg/hour as the initial management [12]; b.) Fluid bolus 20 ml/kg for 2 h, then 2–3 ml/kg/hour in the first 24 h [20]; c.) Isotonic crystalloid > 500 ml/hour for the first 12–24 h [11]. If the RCTs did not report the fluid infusion rate in the study protocols, the crystalloid fluid administration should be greater than 4000 ml in the first 24 h [29]; (3) Comparisons: non-aggressive intravenous fluid resuscitation, defined as a.) Fluid administration at a rate lower than 10 ml/kg/hour; b.) Fluid bolus 10 ml/kg for 2 h; then, 1.5 ml/kg/hour in the first 24 h or c.) Isotonic crystalloid < 500 ml/hour for the first 12–24 h. If the RCTs did not report the fluid infusion rate in the study protocols, the crystalloid fluid administration should be less than 4000 ml in the first 24 h [29]; (4) Primary outcome: all-cause mortality. Other secondary outcomes, such as the rate of clinical improvement (based on the objective parameters, including systemic inflammatory response syndrome (SIRS) subsides and time period therefore, decrease in hematocrit (Hct), blood urea nitrogen (BUN) and creatinine from baseline, and subjective measurements, including decrease in epigastric pain degree, assessed by visual analogue scale (VAS) and tolerance of oral nutrition within 48 h) in non-severe AP and the changes of Acute Physiology and Chronic Health Evaluation II (APACHE II) scores, Sequential Organ Failure Assessment (SOFA) scores and Multiple Organ Dysfunction (MOD) scores for severe AP [30–32], fluid-related complications, such as abdominal compartment syndrome, pulmonary/peripheral edema and any sign of volume overload (e.g., rapid weight gain, incident ascites or jugular vein engorgement), as defined by previous guidelines [20, 33], sepsis, acute respiratory failure, acute kidney injury, pancreatic necrosis, SIRS subsiding within 48 h [34, 35], SIRS persisting > 48 h [34, 35], persistent organ failure (any organ failure > 48 h), defined by the revised Atlanta classification [1] and total hospitalization days were also evaluated if they were reported in the included studies. Specifically, decrease in epigastric pain may be related to better prognosis of AP and better subjective perception of quality of life for patients [7, 36]. In addition, we included APACHE II score changes as the clinical prognosis parameter, based on the suggestions of the ACG guidelines [37], and the prediction performance of poor outcome in severe AP has been validated [37, 38]. We also evaluated the Hct and BUN changes within 48 h [39, 40], because these parameters have been considered as surrogate markers for successful hydration for AP [11]; (5) Designs: RCTs. In cases of disagreement over study selection, the senior author (SCS) made the final decision.

### Data extraction and risk-of-bias assessment

The data extraction and risk-of-bias assessment were performed by two independent reviewers (XWL and CHW). Data extracted included the study (e.g., first author with publication year and study period), participants (e.g., mean age and sex proportion, SIRS on admission and BUN levels) and intervention/comparison (e.g., fluid infusion protocols). We extracted data for the event number and mean with standard deviation (SD) in the intervention and comparison groups for categorical and continuous outcomes, respectively. In RCTs not reporting the SD for changes from baseline in continuous variables, we imputed a change-from-baseline SD using a correlation coefficient approach [41]. We used the Cochrane Collaboration's ROB tool 2.0, addressing the critical domains of randomization process, deviations from intended interventions, missing outcome data, measurement of the outcome, selection of the reported result and overall bias, to evaluate the methodological quality of the included RCTs [42]. Any disagreements between the two reviewers were resolved by the senior author (SCS).

### Data synthesis and statistical analysis

We used Review Manager Version 5.3 (Copenhagen: The Nordic Cochrane Centre, The Cochrane Collaboration, 2014) to conduct a random-effects meta-analysis due to the expected clinical heterogeneity among the included RCTs [43]. Considering the heterogeneity with regard to disease severity among trial participants with AP in the RCTs, we separately calculated the pooled risk ratio (RR) and mean difference (MD) with a 95% confidence interval (CI) for categorical and continuous outcomes, respectively, for severe and non-severe AP. Where multiple scales were employed to measure the same continuous outcome, we used the standardized mean difference (SMD) to express the results. The *I*^2^ statistic was used to determine the extent of statistical heterogeneity among the included RCTs, and a value > 50% was considered as significant heterogeneity [44]. Funnel plots were to be constructed to visually examine for the presence of publication bias if there were at least 10 RCTs included in the meta-analysis [45].

### Subgroup analysis

We assessed the heterogeneity of intravenous hydration effects on the primary and secondary outcomes of study interest based on the trial-level subgroups, including study countries (e.g., Asian or non-Asian), and mean or median age (e.g., < 50 or > 50 years).

### Sensitivity analyses

Recent practice guidelines from AGA and the expert consensus of the ACG Acute Pancreatitis Task Force have highlighted the importance of goal-directed fluid therapy for AP, defined as the titration of intravenous fluids to specific clinical and biochemical perfusion targets (e.g., heart rate, mean arterial pressure, central venous pressure, urine output, BUN and Hct levels) [7, 21]. We therefore replicated all analyses by including only RCTs with goal-directed fluid therapy as a sensitivity analysis to determine the robustness of our results.

### Certainty of evidence of the study outcomes

Two independent reviewers (JWD and CHW) evaluated the CoE for each study outcome based on the Grading of Recommendations Assessment, Development and Evaluation (GRADE) criteria [46]. Any discrepancy between the review authors was resolved by discussion with the senior author (SCS).

## Results

### Characteristics of included studies

We retrieved a total of 239 records from the different databases, plus 7 records from additional reviews found on the reference lists of the included studies, previous review articles and published guidelines regarding AP. The study selection flowchart is presented in Additional file 1: Appendix Fig. S1, and the reasons for exclusion of records after full-text review are presented in Additional file 1: Appendix Table S3. We finally included 9 RCTs (severe AP: 2 RCTs; non-severe AP: 7 RCTs), covering a total of 953 trial participants in this systematic review and meta-analysis [26, 47–54]. We found both of the included RCTs focusing on severe AP were from China [47, 48], while 3, 1, 1, 1 and 1 of the included studies focusing on non-severe AP were from China, Thailand, Mexico, USA and multiple countries, respectively [26, 49–54]. Other important study characteristics of the included RCTs are shown in Table 1 and Additional file 1: Appendix Table S4.

**Table 1:** Study characteristics

First author (Ref)	Country	Study period	Severity^a^	Age, mean years ± SD		Male, n (%)		Participants, n (%)	
				Aggressive	Non-aggressive	Aggressive	Non-aggressive	Aggressive	Non-aggressive
de‑Madaria [26]	India, Italy, Mexico, Spain	2020/05–2021/09	Mild	56 ± 18	57 ± 17	54 (44.3)	68 (53.5)	122	127
Angsubhakorn [51]	Thailand	2019/08–2020/10	Mild	46 ± 15	45 ± 15	18 (81.8)	16 (72.7)	22	22
Liu [52]	China	2019/01–2020/06	Mild to moderately severe	45 ± 13	47 ± 12	36 (75)	44 (63.8)	48	69
Wang [53]	China	2017/06–2019/06	Mild	43 ± 1	42 ± 1	29 (55.8)	28 (53.8)	52	52
Cuéllar [50]	Mexico	2015/05–2016/10	Mild to moderately severe	37 ± 16	39 ± 15	13 (30.2)	18 (40)	43	45
Li [54]	China	2016/01–2017/12	Mild	44 ± 10	46 ± 13	36 (72)	27 (54)	50	50
Buxbaum [49]	U.S	2013/04–2015/11	Mild	44 ± 14	45 ± 12	21 (77.8)	24 (72.3)	27	33
Mao [48]	China	2006/09–2008/12	Severe	50 ± 15	49 ± 10	34 (60.7)	36 (61)	56	59
Mao [47]	China	2001/03–2007/12	Severe	51 ± 14	50 ± 12	N/A	N/A	36	40

First author (Ref)	Initial fluid protocol		Goal-directed therapy	Fluid volume on day 1 after trial entry	
	Aggressive	Non-aggressive		Aggressive	Non-aggressive
de‑Madaria [26]	20 ml/kg bolus then 3 ml/kg/hr	10 ml/kg bolus then 1.5 ml/kg/hr	Yes	5400 ml	3310 ml
Angsubhakorn [51]	20 ml/kg bolus then 3 ml/kg/hr	10 ml/kg bolus then 1.5 ml/kg/hr	Yes	4886 ml	3985 ml
Liu [52]	15 ml/kg bolus then 5 ml/kg/hr after vital sign stable	10 ml/kg bolus then 5 ml/kg/hr after vital signs stable	Yes	3618 ml	3310 ml
Wang [53]	20 ml/kg bolus then 3 ml/kg/hr for 1 week	10 ml/kg bolus then 1.5 ml/kg/hr for 1 week	No	N/A	N/A
Cuéllar [50]	20 mL/kg bolus, then 3 mL/kg/hr for the first 24 h and then 30 mL/kg for the next 24 h	1.5 mL/kg/ hr for the first 24 h and 30 mL/kg during the next 24 h	No	~ 6400 ml^b^	~ 2795 ml^b^
Li [54]	20 ml/kg bolus then 3 ml/kg/hr	10 ml/kg bolus then 1.5 ml/kg/hr	Yes	N/A	N/A
Buxbaum [49]	20 ml/kg bolus then 3 ml/kg/hr	10 ml/kg bolus then 1.5 ml/kg/hr	Yes	5600 ml	3900 ml
Mao [48]	As reference^c^	As reference^c^	Yes	4805 ml^d^	3883 ml^d^
Mao [47]	10–15 ml/kg/hr	5–10 ml/kg/hr	Yes	6855 ml^d^	5841 ml^d^

^a^Severity, severity of the acute pancreatitis

^b^Calculated from the given fluid protocol

^c^Fluid protocol of Mao et al. 2010: H0 × TBW (kg) × 5% = Hg × V1; V1 = (H0/Hg) × TBW × 5% (Liters); H0: Hematocrit on admission; Hg: Goal hematocrit in unit time; TBW: total body weight (kg); V1: Amount of fluid administered to meet the goal HCT; V = V1 + (2.5 L/24 h × unit time (h)). Goal-HCT within 48 h was 34% and 35% in rapid hemodilution and slow hemodilution, respectively

^d^This study followed the mixed fluid type protocol (the ratio of crystalloid and colloid volumes: 2–3:1) as the intravenous hydration for acute pancreatitis. Only lists the amount of crystalloid fluid

### Risk of bias of included studies

We judged only 1 study with 249 participants to have a low risk of bias in all domains [26], and 8 studies as having non-low risk of bias (either “some concerns” or “high risk of bias” in at least one domain). The greatest sources of bias were unclear randomization process and the possibility of selective reporting of results (Additional file 1: Appendix Table S5).

### Primary outcome: all-cause mortality

**Fig. 1:**
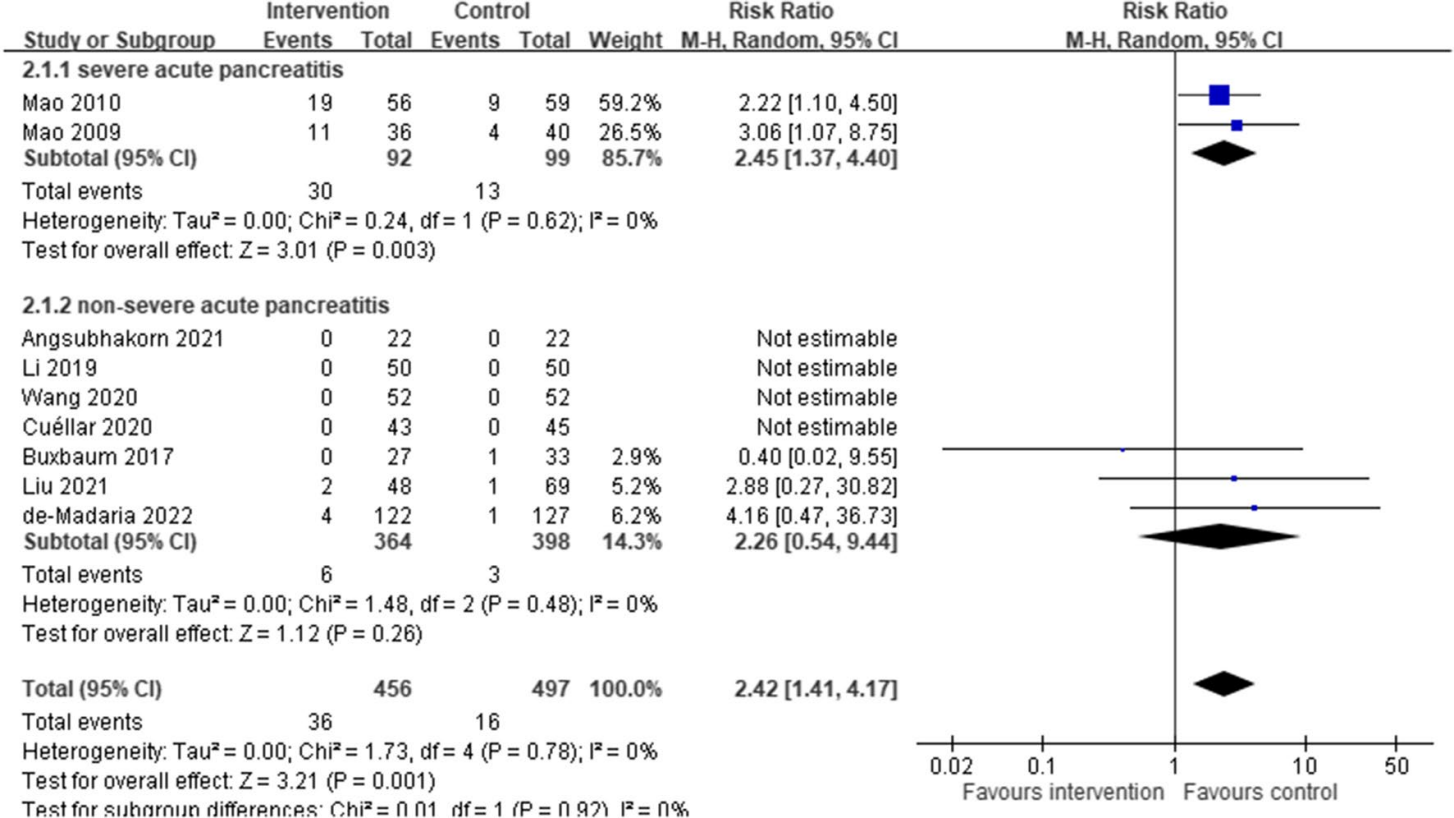
Mortality risk, comparing aggressive (intervention) and non-aggressive (control) hydration protocols for acute pancreatitis

A meta-analysis of 9 RCTs with a total of 953 participants with AP revealed an increased risk of mortality in the aggressive intravenous hydration group, compared to the non-aggressive intravenous hydration group (pooled RR: 2.42, 95% CI: 1.41, 4.17; *I*^2^: 0%) (Fig. 1). However, the significantly increased risk of mortality in the aggressive intravenous hydration group was only observed in severe AP (2 RCTs; 191 participants; pooled RR: 2.45, 95% CI: 1.37, 4.40; *I*^2^: 0%), while in non-severe AP the results remained similar but did not reach statistical significance (7 RCTs, of which only 3 contributed mortality events; 762 participants; pooled RR: 2.26, 95% CI: 0.54, 9.44; *I*^2^: 0%).

### Secondary outcomes: fluid-related complications, clinical improvement, sepsis, acute respiratory failure, acute kidney injury, pancreatic necrosis, SIRS subsiding within 48 h, SIRS persisting > 48 h, persistent organ failure, BUN changes within 48 h, Hct changes within 48 h, and total hospitalization days

**Fig. 2:**
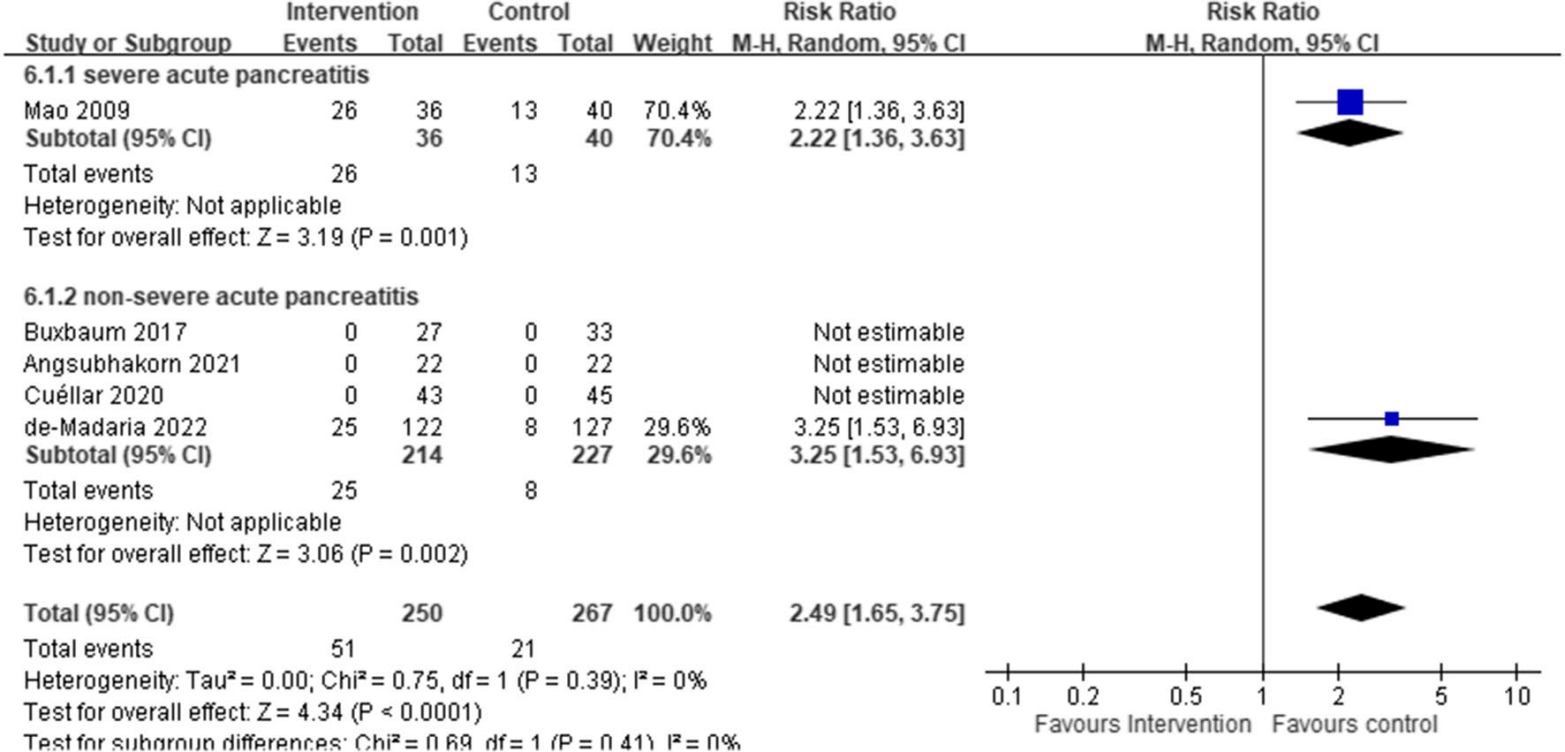
Fluid-related complication risk, comparing aggressive (intervention) and non-aggressive (control) hydration protocols for acute pancreatitis

A meta-analysis of 5 RCTs (of which only 2 contributed outcome events) with a total of 517 participants with AP revealed an increased risk of fluid-related complications in the aggressive intravenous hydration group, compared to the non-aggressive intravenous hydration group (pooled RR: 2.49, 95% CI: 1.65, 3.75; *I*^*2*^: 0%) (Fig. 2). In addition, an increased risk of fluid-related complications in the aggressive intravenous hydration group was found both in severe AP (1 RCTs; 76 participants; RR: 2.22, 95% CI: 1.36, 3.63; *I*^*2*^: not applicable), and in non-severe AP (4 RCTs; 441 participants; pooled RR: 3.25, 95% CI: 1.53, 6.93; *I*^2^: not applicable).

**Fig. 3:**
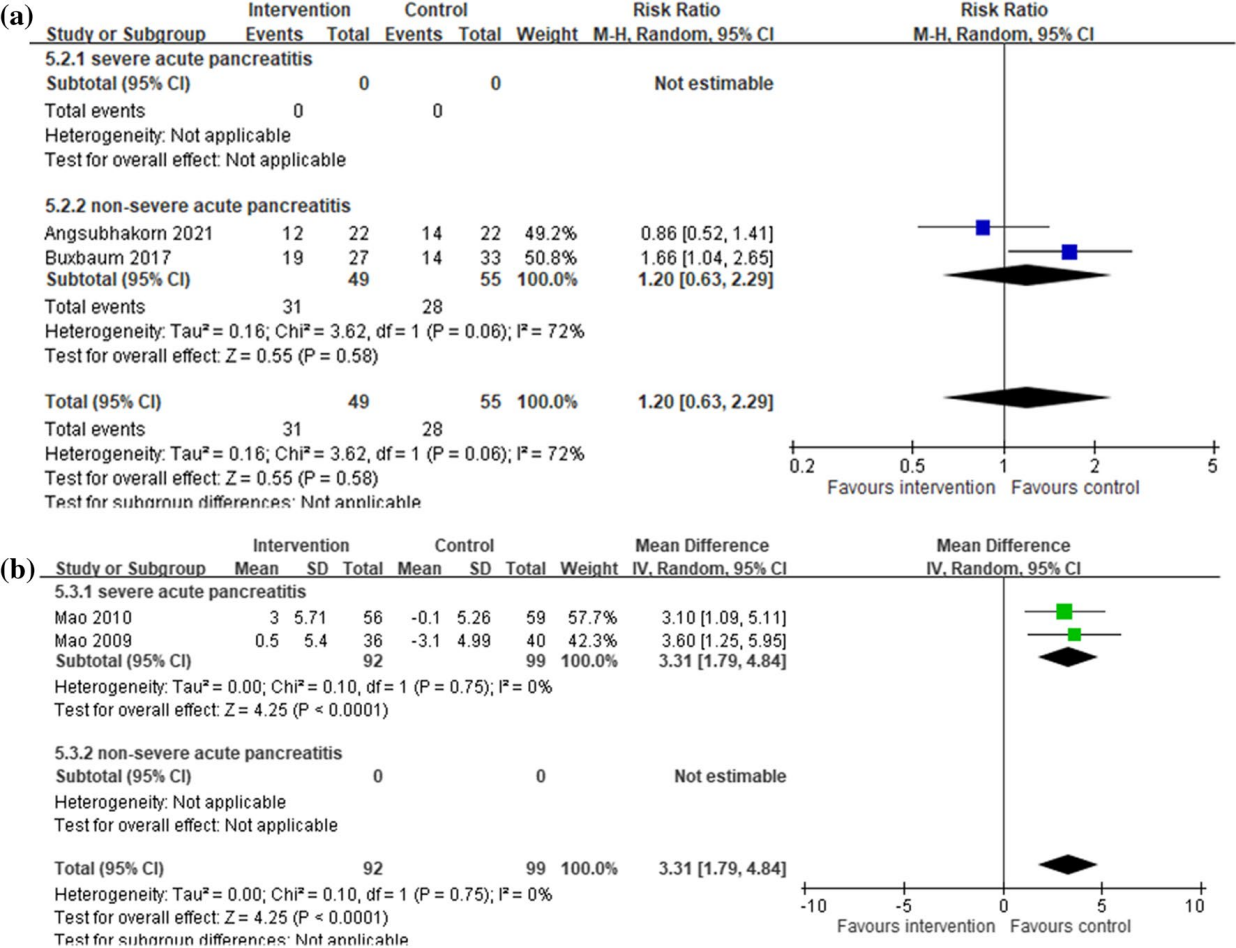
**a** Clinical improvement, comparing aggressive (intervention) and non-aggressive (control) hydration protocols for acute pancreatitis **b** APACHE II score changes within 48 h, comparing aggressive (intervention) and non-aggressive (control) hydration protocols for acute pancreatitis

A meta-analysis of 2 RCTs with a total of 104 participants with non-severe AP revealed no additional clinical improvement in the aggressive intravenous hydration group, compared to the non-aggressive intravenous hydration group (pooled RR: 1.20, 95% CI: 0.63, 2.29; *I*^2^: 72%) (Fig. 3a). None of the included RCTs reported the changes of SOFA or MOD scores. For severe AP, results from the meta-analyses indicated significantly greater changes in APACHE II scores for the aggressive vs. the non-aggressive intravenous hydration group (2 RCTs, 191 participants; pooled MD: 3.31, 95% CI: 1.79, 4.84; *I*^2^: 0%) (Fig. 3b). Another meta-analysis of 3 RCTs with a total of 440 participants with AP revealed an increased risk of sepsis in the aggressive intravenous hydration group, compared to the non-aggressive intravenous hydration group (pooled RR: 1.44, 95% CI: 1.15, 1.80; *I*^2^: 0%) (Additional file 1: Appendix Fig. S2). However, an increased risk of sepsis in the aggressive intravenous hydration group was only found in severe AP (2 RCTs; 191 participants; RR: 1.44, 95% CI: 1.15, 1.80; *I*^2^: 0%), but not in non-severe AP (1 RCTs; 249 participants; pooled RR: 1.73, 95% CI: 0.42, 7.10; *I*^2^: not applicable).

A meta-analysis of 3 RCTs with a total of 442 participants with AP revealed an increased risk of acute respiratory failure in the aggressive intravenous hydration group, compared to the non-aggressive intravenous hydration group (pooled RR: 1.49, 95% CI: 1.18, 1.89; *I*^2^: 0%) (Additional file 1: Appendix Fig. S3). However, an increased risk of acute respiratory failure in the aggressive intravenous hydration group was only found with severe AP (1 RCT; 76 participants; RR: 1.45, 95% CI: 1.14, 1.85; *I*^2^: not applicable), but not with non-severe AP (2 RCTs; 366 participants; pooled RR: 2.46, 95% CI: 0.85, 7.15; *I*^2^: 0%).

No RCTs focusing on severe AP reported acute kidney injury, pancreatic necrosis, SIRS subsiding within 48 h, SIRS persisting > 48 h or persistent organ failure. For those with non-severe AP, results from the meta-analyses did not indicate significant differences between the aggressive and non-aggressive intravenous hydration groups with regard to acute kidney injury (3 RCTs, 441 participants; pooled RR: 0.83, 95% CI: 0.32, 2.16; *I*^2^: 0%), pancreatic necrosis (2 RCTs, 337 participants; pooled RR: 1.82, 95% CI: 0.92, 3.59; *I*^2^: 0%), SIRS subsiding within 48 h (4 RCTs, 441 participants; pooled RR: 1.09, 95% CI: 0.70, 1.70; *I*^2^: 0%), SIRS persisting > 48 h (3 RCTs, 348 participants; pooled RR: 1.04, 95% CI: 0.50, 2.16; *I*^2^: 30%) or persistent organ failure (5 RCTs, of which only 4 contributed outcome events, 558 participants; pooled RR: 1.34, 95% CI: 0.65, 2.79; *I*^2^: 22%) (Additional file 1: Appendix Fig. S4–S8).

A meta-analysis of 2 RCTs with a total of 191 participants with severe AP revealed no significant differences in Hct changes within 48 h in the aggressive intravenous hydration group, compared to the non-aggressive intravenous hydration group (pooled MD: − 4.41, 95% CI: − 15.97, 7.16; *I*^2^: 100%). No RCTs focusing on severe AP reported BUN changes within 48 h or total hospitalization days. For non-severe AP, we did not find significant differences between the aggressive and non-aggressive intravenous hydration protocols as regards BUN changes within 48 h (2 RCTs, 104 participants; pooled MD: − 1.84, 95% CI: − 3.76, 0.08; *I*^2^: 0%), Hct changes within 48 h (2 RCTs, 104 participants; pooled MD: − 1.25, 95% CI: − 3.90, 1.41; *I*^2^: 71%) and total hospitalization days (6 RCTs, 673 participants; pooled MD: − 0.43, 95% CI: − 2.03, 1.17; *I*^2^: 98%) (Additional file 1: Appendix Figs. S9–S11).

### Subgroup analyses

These results from the subgroup analyses of primary (Additional file 1: Appendix Table S6) and secondary outcomes (Additional file 1: Appendix Table S7–S19), comparing aggressive and non-aggressive intravenous hydration protocols for severe and non-severe AP, were generally similar to those from the main analysis. For example, compared with non-aggressive intravenous hydration protocols, the increased mortality risk associated with aggressive intravenous hydration protocols was also observed in participants with severe AP from Asian countries (2 RCTs, 191 participants; pooled RR: 2.45, 95% CI: 1.37, 4.40; *I*^2^: 0%), while inconclusive results were observed for non-severe AP.

### Sensitivity analyses

In the sensitivity analyses, we included 7 RCTs (severe AP: 2 RCTs; non-severe AP: 5 RCTs) examining intravenous hydration protocols based on goal-directed fluid therapy after the initial fluid resuscitation therapy. The results also indicated an increased risk of mortality in the severe AP group receiving aggressive intravenous hydration (2 RCTs, 191 participants; pooled RR: 2.45, 95% CI: 1.37, 4.40; *I*^2^: 0%) (Additional file 1: Appendix Fig. S12), and increased risks of fluid-related complications in both the severe (1 RCT, 76 participants; pooled RR: 2.22, 95% CI: 1.36, 3.63; *I*^2^: not applicable) and non-severe AP group (3 RCTs, of which only 1 contributed outcome events, 353 participants; pooled RR: 3.25, 95% CI: 1.53, 6.93; *I*^2^: not applicable) receiving aggressive intravenous hydration, compared to the non-aggressive intravenous hydration group (Additional file 1: Appendix Fig. S13). Other secondary outcomes from sensitivity analyses were consistent with those from the main analyses (Additional file 1: Appendix Figs. S12–S25 and Tables S20–33).

### GRADE assessment

Since there was a serious risk of bias and imprecise results due to the small sample sizes in the included RCTs, we judged the CoE by the GRADE criteria for our primary and secondary outcomes to be low to very low (Additional file 1: Appendix Table S34).

## Discussion

In this systematic review and meta-analysis of 9 RCTs, the mortality risk from selecting aggressive intravenous hydration protocols over non-aggressive intravenous hydration protocols for fluid resuscitation therapy significantly increased 2.45-fold in severe AP, while in non-severe AP the results remained similar but did not reach statistical significance. This finding may be explained by our analyses of secondary outcomes, which indicated that aggressive intravenous hydration protocols did not decrease APACHE II scores in severe AP, or improve the clinical conditions in non-severe AP. In addition, aggressive intravenous hydration protocols increased the fluid-related complication risk in severe and non-severe AP by 2.22–3.25 times. Our findings suggested that aggressive intravenous hydration protocols should not be recommended for fluid therapy in severe and non-severe AP and may serve as an important reference not only for clinical practitioners but also for future practice guideline makers.

Determining an appropriate fluid therapy strategy for AP, especially as regards infusion rate, is critical but remains controversial. Previous animal studies have suggested that aggressive fluid therapy could improve splenic blood flow, correct pancreatic hypoperfusion and ultimately reduce pancreatic damage and mortality [55, 56], but may cause higher central venous pressure, leading to side effects of interstitial edema without significant changes in mean arterial pressure [57]. Conflicting treatment effects from aggressive intravenous fluid therapy have been found in previous RCTs and observational studies in patients with AP [23, 24, 29, 43, 44]. Nonetheless, ACG guidelines recommend aggressive intravenous hydration (defined as 250–500 ml per hour of isotonic crystalloid solution), to be administered early to most patients with AP [11], but the AGA guidelines and the ACG Acute Pancreatitis Task Force consensus make no recommendations on the hydration infusion rate [7, 21]. Our findings did not support the practice guidelines of the ACG or AISP and extended the knowledge gaps in the ACG/Acute Pancreatitis Task Force consensus. Our meta-analysis indicated a greater than twofold increased risk of mortality and fluid overload associated with aggressive intravenous hydration protocols in severe AP cases, and a similarly increased risk of fluid overload in non-severe AP cases.

AP induces interstitial edema and increases inter-capillary distance and therefore, leads to focal ischemia [14, 45]. In severe AP, in addition to interstitial edema, the production of free radicals and vasoconstriction of small arterioles with inflammatory cells adhering to the endothelial cells all contribute to a cytokine cascade [55, 56, 58], leading to multi-organ dysfunction syndrome, which is associated with higher mortality [13, 57, 59, 60]. Fluid overload itself may worsen the natural course of severe AP, with the accumulating fluid leading to abdominal compartment syndrome which then further compromises the kidneys, lungs and peritoneal viscera, which also potentially increases the risk of multi-organ failure and mortality [59, 61]. The aforementioned mechanisms could explain the poorer results in several of our secondary outcomes, such as APACHE II changes, acute respiratory failure and sepsis, that may derive from aggressive vs. non-aggressive intravenous hydration protocols in severe AP. Overall, aggressive intravenous hydration is not suggested for patients with severe AP because it not only increases the risk of mortality, but also the risk of fluid overload.

In contrast to patients with severe AP, this meta-analysis did not find a significantly increased mortality risk or clinical improvement rate from aggressive intravenous hydration in patients with non-severe AP. Mild AP is mostly self-limiting, causes only local inflammation and usually resolves within one week [10], and therefore is unlikely to increase mortality, affect the clinical progression of pancreatitis or cause multi-organ failure due to fluid-related complications. Our analyses of several secondary outcomes such as clinical improvement, sepsis, acute respiratory failure, acute kidney injury, pancreatic necrosis, BUN changes within 48 h, Hct changes within 48 h, SIRS subsiding within 48 h, SIRS persisting > 48 h, and persistent organ failure further supported this viewpoint. Our results were also consistent with previous large cohort studies [40, 62]. Specifically, the clinical improvement outcome included the subsiding of epigastric pain, which reflected overall clinical improvement and integrated the patient’s subjective symptoms, and our finding may imply that patients with AP receiving aggressive intravenous hydration, compared to non-aggressive intravenous hydration, may not achieve better quality of life in terms of pain relief [63]. Taken together, our findings suggest that aggressive intravenous hydration is not recommended for patients with non-severe AP, because it may increase the fluid overload risk while not actually improving clinical conditions.

Early goal-directed therapy has recently been suggested to titrate the intravenous fluid to specific clinical and biochemical targets of perfusion (e.g., heart rate, mean arterial pressure, central venous pressure, urine output, BUN, and Hct). The treatment benefits of goal-directed therapy in AP, compared to non-targeted therapy, remain inconsistent according to the findings of previous RCTs, but the AGA guidelines included a conditional recommendation suggesting the use of goal-directed fluid therapy versus other methods [7]. In this systematic review and meta-analysis, we replicated the analyses by only including RCTs using early goal-directed therapy in intravenous hydration protocols, and the results remained consistent to those from the main analyses. We found that aggressive intravenous hydration protocols were still associated with a higher risk of mortality and fluid-related complications in AP, even with the goal-directed fluid strategy. Our findings provided further robust evidence supporting the use of conservative intravenous hydration protocols for AP [7].

### Strength and limitations

To the best of our knowledge, this is the first and most comprehensive systematic review and meta-analysis of RCTs that examine and compare outcomes between aggressive and non-aggressive intravenous hydration protocols for fluid therapy to treat severe and non-severe AP. In contrast to previous reviews which included both RCTs and cohort studies [22–24, 64], we included only RCTs in order to achieve more homogeneity between studies, and in order to provide the highest quality of evidence to address the specific research questions of clinicians, researchers, and health policy makers [65]. More importantly, we critically examined every potential record to ensure eligibility of the trials for our review. For example, we excluded one RCT by Wu et al. which was frequently included in previous meta-analyses on this topic, because the comparison of this RCT did not receive the non-aggressive intravenous hydration protocol [22, 23, 66]. Furthermore, we performed more pre-specified subgroup analyses to examine the heterogeneity of treatment effects, and the results from these subgroup analyses were generally consistent with our main analysis. Finally, we replicated all analyses by including only RCTs with goal-directed fluid therapy for AP, as suggested by the AGA and ACG Acute Pancreatitis Task Force since 2018 [2], and these findings were also consistent with our main analysis. Overall, our findings highlighted the clinical importance of a more conservative intravenous hydration therapy.

Several limitations should be noted before final interpretation of our findings. First, due to the limited number of included trial participants, the statistical power to detect significant differences in the secondary outcomes and the results from subgroup analyses may be suboptimal. However, our review did include four Asian RCTs and one multi-country RCT that had never been included in previous reviews, thus providing greater precision around estimates of treatment effects. Second, no individual-level data were obtained to examine the heterogeneity of treatment effects with different etiologies of AP [67, 68]. However, our included RCTs had participants with various etiologies of AP, so our findings may be applicable to the overall AP population. Third, the included RCTs did not report the total volumes of resuscitation fluid for the trial participants during hospitalization, so the actual volume differences between the aggressive and non-aggressive intravenous hydration groups remain unclear. Fourth, we could not investigate publication bias since we only included 9 RCTs in this meta-analysis. Finally, we judged the CoE of all study outcomes as low to very low, largely due to methodological concerns and small sample sizes of the included RCTs. We suggest regularly updating systematic reviews to include newly published RCTs on this critical topic.

## Conclusion

This systematic review and meta-analysis of currently existing RCTs indicates that aggressive intravenous hydration protocols increased the mortality risk in severe AP and the fluid-related complication risk in both severe and non-severe AP. Our findings highlighted the clinical importance of a more conservative approach to fluid therapy for AP in order to mitigate excessive risk of avoidable side effects and mortality.

## Supplementary Information

**Additional file 1**. Supplementary tables and figures.

## Data Availability

We present the study data, in the form of tables or appendix files, in this manuscript.

## Abbreviations

ACG: American college of gastroenterology
AGA: American gastroenterological association
AISP: Association for the study of the pancreas
AP: Acute pancreatitis
APACHE II: Acute physiology and chronic health evaluation II
Hct: Hematocrit
BUN: Blood urea nitrogen
CI: Confidence interval
CoE: Certainty of evidence
GRADE: Grading of recommendations assessment, development and evaluation
INPLASY: International platform of registered systematic review and meta-analysis protocols
MD: Mean difference
RCTs: Randomized controlled trials
PRISMA: Preferred reporting items for systematic reviews and meta-analyses
RR: Risk ratio
SD: Standard deviation
SIRS: Systemic inflammatory response syndrome
SMD: Standardized mean difference
SOFA: Sequential organ failure assessment
MOD: Multiple organ dysfunction
