# Supplementary material for: Comparison of clinical outcomes between aggressive and non-aggressive intravenous hydration for acute pancreatitis: a systematic review and meta-analysis
Source: Crit Care. 2023 Mar 22;27:122. doi: 10.1186/s13054-023-04401-0 (PMC10035244; doi:10.1186/s13054-023-04401-0)
Supplement: Supplementary file 1 — Additional file 1. Supplementary tables and figures. [file 13054_2023_4401_MOESM1_ESM.docx]

**Appendix Material**

**Appendix Figure 1.** PRISMA Study Flowchart

**Appendix Figure 2.** Forest plot for sepsis, comparing aggressive (intervention) and non-aggressive (control) protocols for acute pancreatitis

**Appendix Figure 3.** Forest plot for acute respiratory failure, comparing aggressive (intervention) and non-aggressive (control) protocols for acute pancreatitis

**Appendix Figure 4.** Forest plot for acute kidney injury, comparing aggressive (intervention) and non-aggressive (control) protocols for acute pancreatitis

**Appendix Figure 5.** Forest plot for pancreatic necrosis, comparing aggressive (intervention) and non-aggressive (control) protocols for acute pancreatitis

**Appendix Figure 6.** Forest plot for SIRS subsiding within 48 hours, comparing aggressive (intervention) and non-aggressive (control) protocols for acute pancreatitis

**Appendix Figure 7.** Forest plot for SIRS persisting >48 hours, comparing aggressive (intervention) and non-aggressive (control) protocols for acute pancreatitis

**Appendix Figure 8.** Forest plot for persistent organ failure, comparing aggressive (intervention) and non-aggressive (control) protocols for acute pancreatitis

**Appendix Figure 9.** Forest plot for BUN changes within 48 hours, comparing aggressive (intervention) and non-aggressive (control) protocols for acute pancreatitis

**Appendix Figure 10.** Forest plot for Hct changes within 48 hours, comparing aggressive (intervention) and non-aggressive (control) protocols for acute pancreatitis **Appendix Figure 11.** Forest plot for total hospitalization days, comparing aggressive (intervention) and non-aggressive (control) protocols for acute pancreatitis

**Appendix Figure 12.** Forest plot for the sensitivity analysis: Mortality risk, comparing aggressive (intervention) and non-aggressive (control) protocols for acute pancreatitis

**Appendix Figure 13.** Forest plot for the sensitivity analysis: Fluid-related complication risk, comparing aggressive (intervention) and non-aggressive (control) protocols for acute pancreatitis

**Appendix Figure 14.** Forest plot for the sensitivity analysis: Clinical improvement, comparing aggressive (intervention) and non-aggressive (control) protocols for acute pancreatitis

**Appendix Figure 15.** Forest plot for the sensitivity analysis: APACHE II score changes within 48 hours, comparing aggressive (intervention) and non-aggressive (control) protocols for acute pancreatitis

**Appendix Figure 16.** Forest plot for the sensitivity analysis: Sepsis, comparing aggressive (intervention) and non-aggressive (control) protocols for acute pancreatitis

**Appendix Figure 17.** Forest plot for the sensitivity analysis: Acute respiratory failure, comparing aggressive (intervention) and non-aggressive (control) protocols for acute pancreatitis

**Appendix Figure 18.** Forest plot for the sensitivity analysis: Acute kidney injury, comparing aggressive (intervention) and non-aggressive (control) protocols for acute pancreatitis

**Appendix Figure 19.** Forest plot for the sensitivity analysis: Pancreatic necrosis, comparing aggressive (intervention) and non-aggressive (control) protocols for acute pancreatitis

**Appendix Figure 20.** Forest plot for the sensitivity analysis: SIRS subsiding within 48 hours, comparing aggressive (intervention) and non-aggressive (control) protocols for acute pancreatitis

**Appendix Figure 21.** Forest plot for the sensitivity analysis: SIRS persisting >48 hours, comparing aggressive (intervention) and non-aggressive (control) protocols for acute pancreatitis

**Appendix Figure 22.** Forest plot for the sensitivity analysis: Persistent organ failure, comparing aggressive (intervention) and non-aggressive (control) protocols for acute pancreatitis

**Appendix Figure 23.** Forest plot for the sensitivity analysis: BUN changes within 48 hours, comparing aggressive (intervention) and non-aggressive (control) protocols for acute pancreatitis

**Appendix Figure 24.** Forest plot for the sensitivity analysis: Hct changes within 48 hours, comparing aggressive (intervention) and non-aggressive (control) protocols for acute pancreatitis

**Appendix Figure 25.** Forest plot for the sensitivity analysis: Total hospitalization days, comparing aggressive (intervention) and non-aggressive (control) protocols for acute pancreatitis

**Appendix Table 1.** PRISMA Checklist

**Appendix Table 2.** Search strategy

**Appendix Table 3.** Reasons for exclusion of records after full-text review

**Appendix Table 4.** Other study characteristics of included studies

**Appendix Table 5.** RoB

**Appendix Table 6.** Subgroup analyses: Mortality

**Appendix Table 7.** Subgroup analyses: Clinical improvement

**Appendix Table 8.** Subgroup analyses: APACHE II score changes

**Appendix Table 9.** Subgroup analyses: Fluid-related complications

**Appendix Table 10.** Subgroup analyses: Acute respiratory failure

**Appendix Table 11.** Subgroup analyses: Sepsis

**Appendix Table 12.** Subgroup analyses: Acute kidney injury

**Appendix Table 13.** Subgroup analyses: Pancreatic necrosis

**Appendix Table 14.** Subgroup analyses: SIRS subsiding within 48 hours

**Appendix Table 15.** Subgroup analyses: SIRS persisting >48 hours

**Appendix Table 16.** Subgroup analyses: Persistent organ failure

**Appendix Table 17.** Subgroup analyses: BUN changes within 48 hours

**Appendix Table 18.** Subgroup analyses: Hct changes within 48 hours

**Appendix Table 19.** Subgroup analyses: Total hospitalization days

**Appendix Table 20.** Subgroup analyses of sensitivity analyses: Mortality

**Appendix Table 21.** Subgroup analyses of sensitivity analyses: Clinical improvement

**Appendix Table 22.** Subgroup analyses: APACHE II score changes

**Appendix Table 23.** Subgroup analyses of sensitivity analyses: Fluid-related complications

**Appendix Table 24.** Subgroup analyses of sensitivity analyses: Acute respiratory failure

**Appendix Table 25.** Subgroup analyses of sensitivity analyses: Sepsis

**Appendix Table 26.** Subgroup analyses of sensitivity analyses: Acute kidney injury

**Appendix Table 27.** Subgroup analyses of sensitivity analyses: Pancreatic necrosis

**Appendix Table 28.** Subgroup analyses of sensitivity analyses: SIRS subsiding within 48 hours

**Appendix Table 29.** Subgroup analyses of sensitivity analyses: SIRS persisting >48 hours

**Appendix Table 30.** Subgroup analyses of sensitivity analyses: Persistent organ failure

**Appendix Table 31.** Subgroup analyses of sensitivity analyses: BUN changes within 48 hours

**Appendix Table 32.** Subgroup analyses of sensitivity analyses: Hct changes within 48 hours

**Appendix Table 33.** Subgroup analyses of sensitivity analyses: Total hospitalization days

**Appendix Table 34.** GRADE assessment on study outcomes from included RCTs

Records from Cochrane Library, N= 72

Records from Embase, N= 122

Records from PubMed, N= 45

Other records from reference lists, previous review articles and treatment guidelines, N= 7

Records screened, N= 246

1. Duplicate records, N= 85
2. Not related to the research questions, N= 115

Full-text articles assessed for eligibility, N= 46

Full-text articles excluded:

1. Not RCTs, N= 25
2. Not focusing on adults with acute pancreatitis, N= 1
3. Not focusing on aggressive vs. non-aggressive hydration protocols, N= 11
4. No data of mortality, N=0

Studies included in qualitative synthesis, N= 9

Studies included in quantitative synthesis (meta-analysis), N= 9

**Appendix Figure 1.** PRISMA Study Flowchart

**
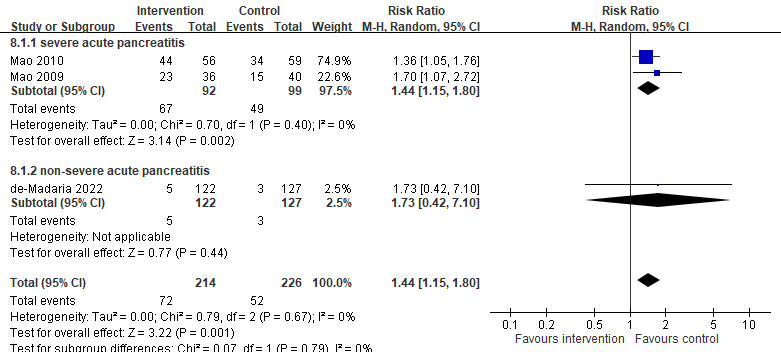
**

**Appendix Figure 2.** Sepsis, comparing aggressive (intervention) and non-aggressive (control) protocols for acute pancreatitis

**
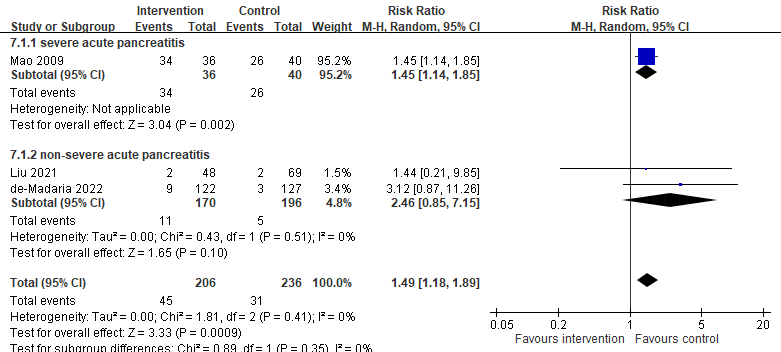
**

**Appendix Figure 3.** Acute respiratory failure, comparing aggressive (intervention) and non-aggressive (control) protocols for acute pancreatitis

**
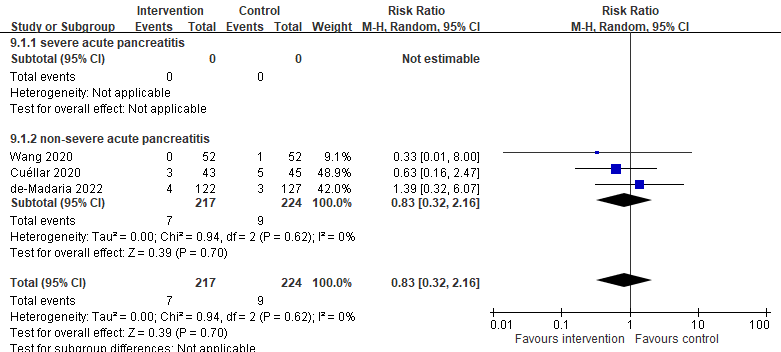
**

**Appendix Figure 4.** Acute kidney injury, comparing aggressive (intervention) and non-aggressive (control) protocols for acute pancreatitis

**
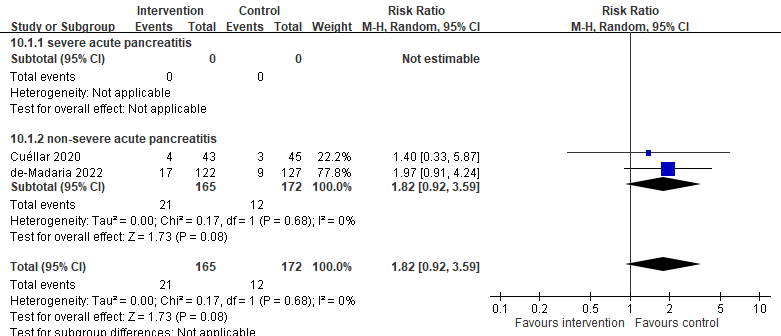
**

**Appendix Figure 5.** Pancreatic necrosis, comparing aggressive (intervention) and non-aggressive (control) protocols for acute pancreatitis


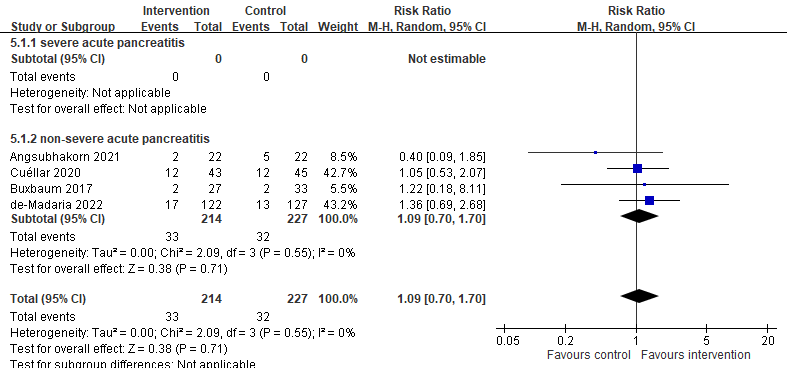


**Appendix Figure 6.** SIRS subsiding within 48 hours, comparing aggressive (intervention) and non-aggressive (control) protocols for acute pancreatitis


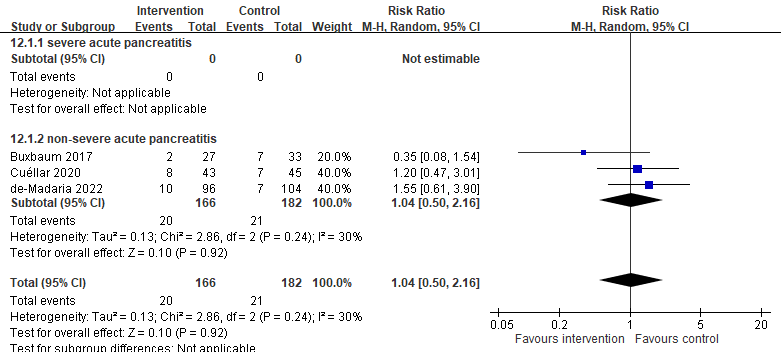


**Appendix Figure 7.** SIRS persisting >48 hours, comparing aggressive (intervention) and non-aggressive (control) protocols for acute pancreatitis


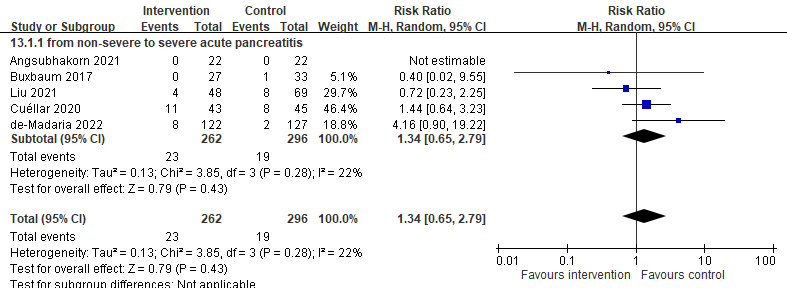


**Appendix Figure 8.** Persistent organ failure, comparing aggressive (intervention) and non-aggressive (control) protocols for acute pancreatitis


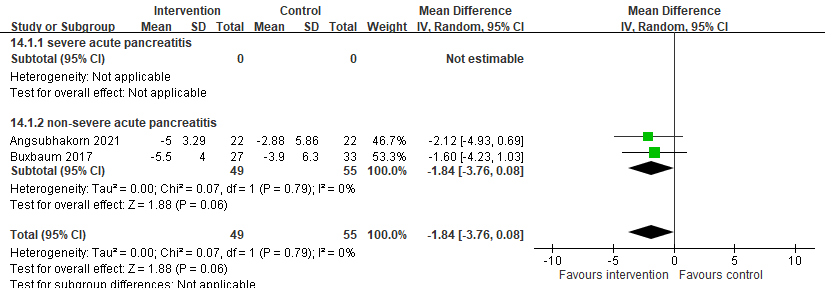


**Appendix Figure 9.** BUN changes within 48 hours, comparing aggressive (intervention) and non-aggressive (control) protocols for acute pancreatitis


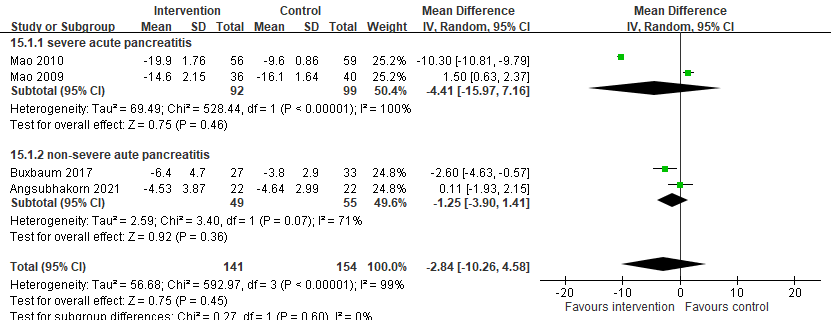


**Appendix Figure 10.** Hct changes within 48 hours, comparing aggressive (intervention) and non-aggressive (control) protocols for acute pancreatitis

**
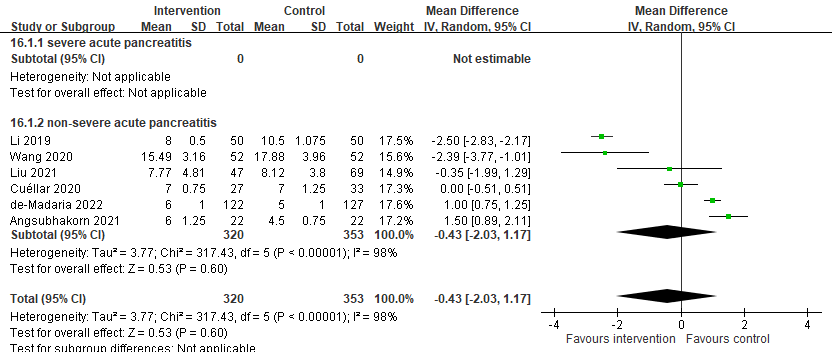
**

**Appendix Figure 11.** Total hospitalization days, comparing aggressive (intervention) and non-aggressive (control) protocols for acute pancreatitis

**
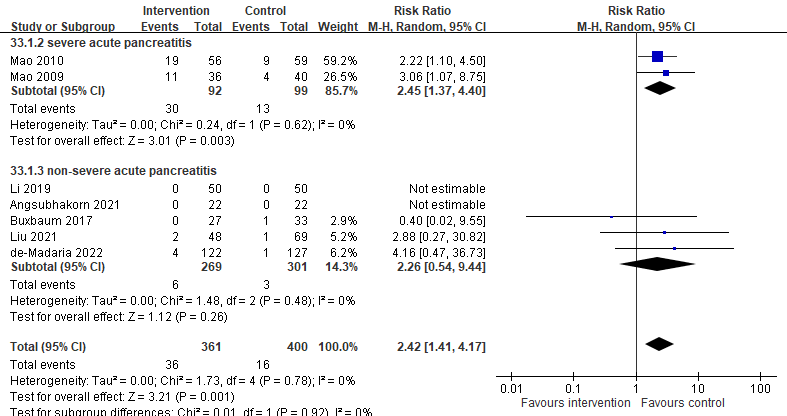
**

**Appendix Figure 12.** Sensitivity analysis: Mortality risk, comparing aggressive (intervention) and non-aggressive (control) protocols for acute pancreatitis

**
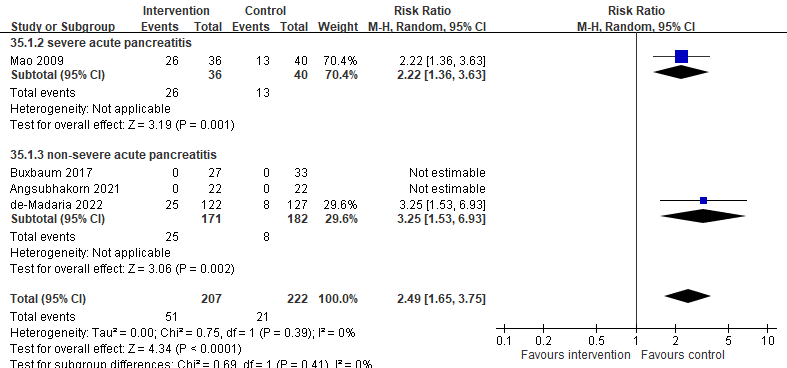
**

**Appendix Figure 13.** Sensitivity analysis: Fluid-related complication risk, comparing aggressive (intervention) and non-aggressive (control) protocols for acute pancreatitis

**
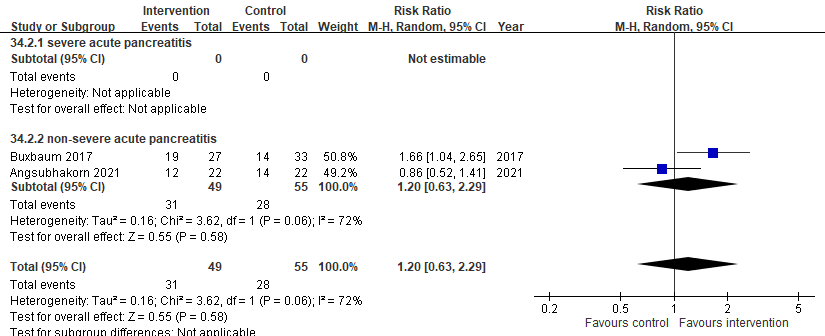
**

**Appendix Figure 14.** Sensitivity analysis: Clinical improvement, comparing aggressive (intervention) and non-aggressive (control) protocols for acute pancreatitis

**
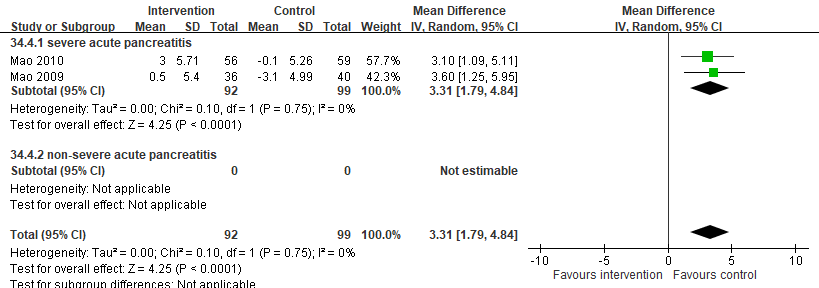
**

**Appendix Figure 15.** Sensitivity analysis: APACHE II score changes within 48 hours, comparing aggressive (intervention) and non-aggressive (control) protocols for acute pancreatitis

**
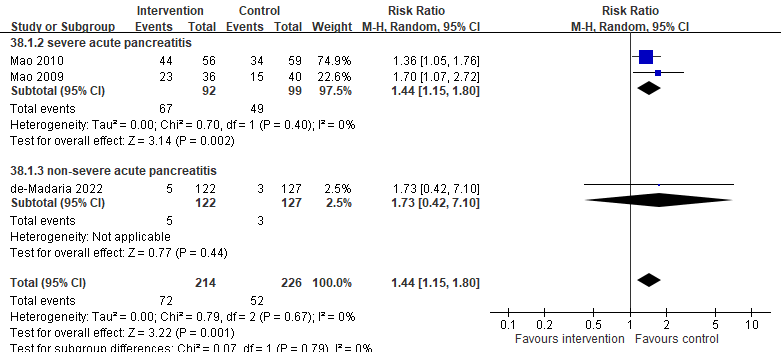
**

**Appendix Figure 16.** Sensitivity analysis: Sepsis, comparing aggressive (intervention) and non-aggressive (control) protocols for acute pancreatitis


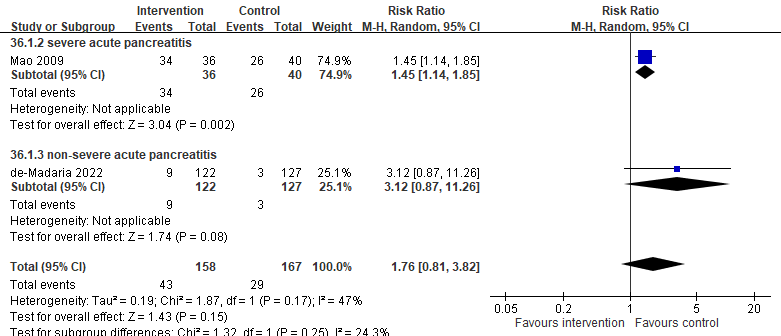


**Appendix Figure 17.** Sensitivity analysis: Acute respiratory failure, comparing aggressive (intervention) and non-aggressive (control) protocols for acute pancreatitis

**
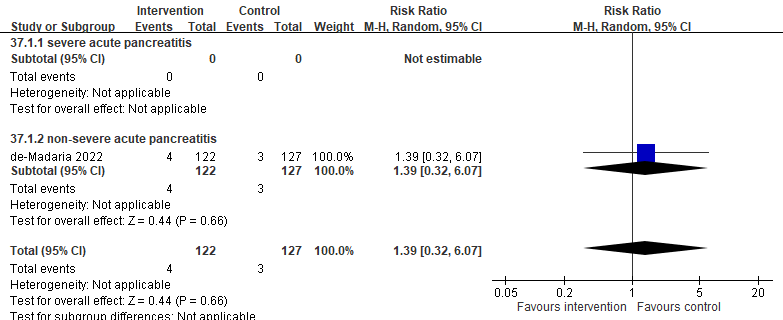
**

**Appendix Figure 18.** Sensitivity analysis: Acute kidney injury, comparing aggressive (intervention) and non-aggressive (control) protocols for acute pancreatitis


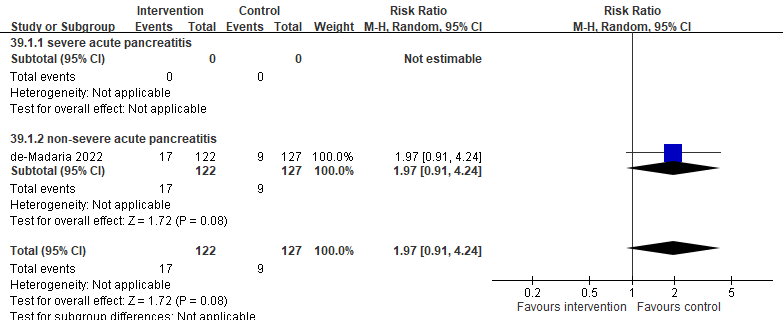


**Appendix Figure 19.** Sensitivity analysis: Pancreatic necrosis, comparing aggressive (intervention) and non-aggressive (control) protocols for acute pancreatitis


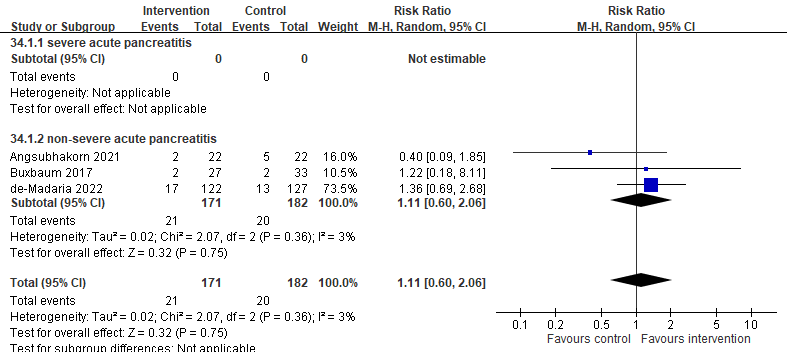


**Appendix Figure 20.** Sensitivity analysis: SIRS subsiding within 48 hours, comparing aggressive (intervention) and non-aggressive (control) protocols for acute pancreatitis


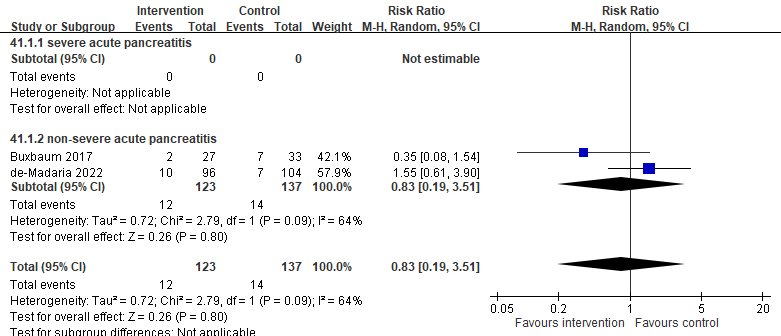


**Appendix Figure 21.** Sensitivity analysis: SIRS persisting >48 hours, comparing aggressive (intervention) and non-aggressive (control) protocols for acute pancreatitis


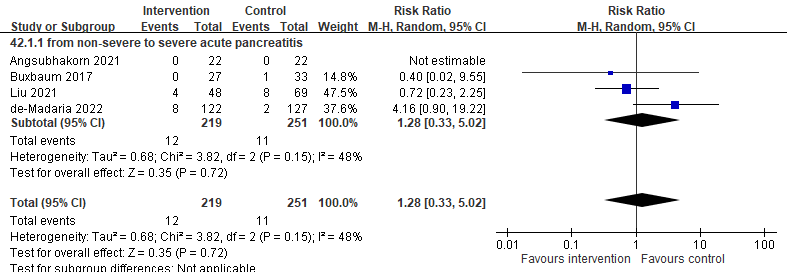


**Appendix Figure 22.** Sensitivity analysis: Persistent organ failure, comparing aggressive (intervention) and non-aggressive (control) protocols for acute pancreatitis


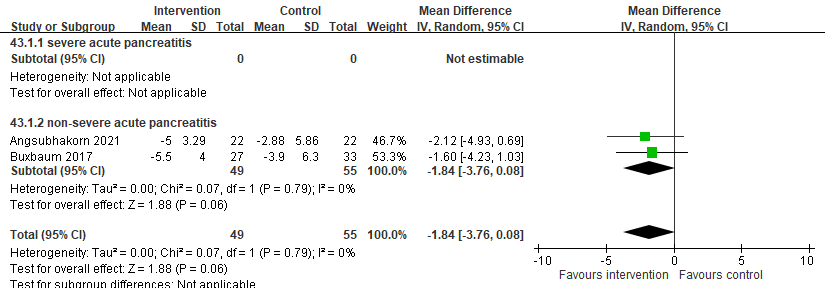


**Appendix Figure 23.** Sensitivity analysis: BUN changes within 48 hours, comparing aggressive (intervention) and non-aggressive (control) protocols for acute pancreatitis


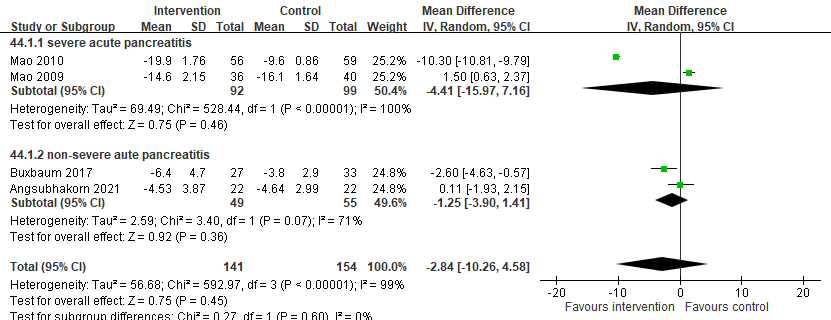


**Appendix Figure 24.** Sensitivity analysis: Hct changes within 48 hours, comparing aggressive (intervention) and non-aggressive (control) protocols for acute pancreatitis

**
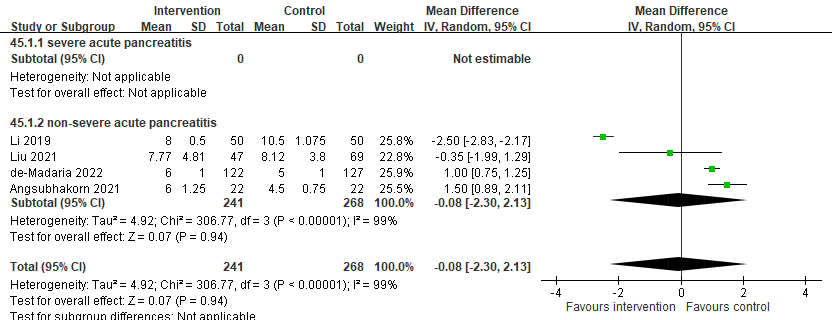
**

**Appendix Figure 25.** Sensitivity analysis: Total hospitalization days, comparing aggressive (intervention) and non-aggressive (control) protocols for acute pancreatitis

| **Appendix Table 1.** PRISMA Checklist | | | |
| --- | --- | --- | --- |
| **Section and Topic** | **Item #** | **Checklist item** | **Location where item is reported** |
| **TITLE** | | |  |
| Title | 1 | Identify the report as a systematic review. | 1 |
| **ABSTRACT** | | |  |
| Abstract | 2 | See the PRISMA 2020 for Abstracts checklist. | 3-4 |
| **INTRODUCTION** | | |  |
| Rationale | 3 | Describe the rationale for the review in the context of existing knowledge. | 5-6 |
| Objectives | 4 | Provide an explicit statement of the objective(s) or question(s) the review addresses. | 6 |
| **METHODS** | | |  |
| Eligibility criteria | 5 | Specify the inclusion and exclusion criteria for the review and how studies were grouped for the syntheses. | 7-8 |
| Information sources | 6 | Specify all databases, registers, websites, organisations, reference lists and other sources searched or consulted to identify studies. Specify the date when each source was last searched or consulted. | 7 |
| Search strategy | 7 | Present the full search strategies for all databases, registers and websites, including any filters and limits used. | 7 |
| Selection process | 8 | Specify the methods used to decide whether a study met the inclusion criteria of the review, including how many reviewers screened each record and each report retrieved, whether they worked independently, and if applicable, details of automation tools used in the process. | 7-8 |
| Data collection process | 9 | Specify the methods used to collect data from reports, including how many reviewers collected data from each report, whether they worked independently, any processes for obtaining or confirming data from study investigators, and if applicable, details of automation tools used in the process. | 9-10 |
| Data items | 10a | List and define all outcomes for which data were sought. Specify whether all results that were compatible with each outcome domain in each study were sought (e.g. for all measures, time points, analyses), and if not, the methods used to decide which results to collect. | 8-9 |
|  | 10b | List and define all other variables for which data were sought (e.g. participant and intervention characteristics, funding sources). Describe any assumptions made about any missing or unclear information. | 8-9 |
| Study risk of bias assessment | 11 | Specify the methods used to assess risk of bias in the included studies, including details of the tool(s) used, how many reviewers assessed each study and whether they worked independently, and if applicable, details of automation tools used in the process. | 10 |
| Effect measures | 12 | Specify for each outcome the effect measure(s) (e.g. risk ratio, mean difference) used in the synthesis or presentation of results. | 10 |
| Synthesis methods | 13a | Describe the processes used to decide which studies were eligible for each synthesis (e.g. tabulating the study intervention characteristics and comparing against the planned groups for each synthesis (item #5)). | 10 |
|  | 13b | Describe any methods required to prepare the data for presentation or synthesis, such as handling of missing summary statistics, or data conversions. | 10 |
|  | 13c | Describe any methods used to tabulate or visually display results of individual studies and syntheses. | 10 |
|  | 13d | Describe any methods used to synthesize results and provide a rationale for the choice(s). If meta-analysis was performed, describe the model(s), method(s) to identify the presence and extent of statistical heterogeneity, and software package(s) used. | 10 |
|  | 13e | Describe any methods used to explore possible causes of heterogeneity among study results (e.g. subgroup analysis, meta-regression). | 10 |
|  | 13f | Describe any sensitivity analyses conducted to assess robustness of the synthesized results. | 11 |
| Reporting bias assessment | 14 | Describe any methods used to assess risk of bias due to missing results in a synthesis (arising from reporting biases). | NA |
| Certainty assessment | 15 | Describe any methods used to assess certainty (or confidence) in the body of evidence for an outcome. | 11 |
| **RESULTS** | | |  |
| Study selection | 16a | Describe the results of the search and selection process, from the number of records identified in the search to the number of studies included in the review, ideally using a flow diagram. | 12 |
|  | 16b | Cite studies that might appear to meet the inclusion criteria, but which were excluded, and explain why they were excluded. | 12 |
| Study characteristics | 17 | Cite each included study and present its characteristics. | Table 1 |
| Risk of bias in studies | 18 | Present assessments of risk of bias for each included study. | Appendix Table 5 |
| Results of individual studies | 19 | For all outcomes, present, for each study: (a) summary statistics for each group (where appropriate) and (b) an effect estimate and its precision (e.g. confidence/credible interval), ideally using structured tables or plots. | Figure 1-3 |
| Syntheses | 20a | For each synthesis, briefly summarise the characteristics and risk of bias among contributing studies. | 12-14 |
|  | 20b | Present results of all statistical syntheses conducted. If meta-analysis was done, present for each the summary estimate and its precision (e.g. confidence/credible interval) and measures of statistical heterogeneity. If comparing groups, describe the direction of the effect. | 12-14 |
|  | 20c | Present results of all investigations of possible causes of heterogeneity among study results. | 15 |
|  | 20d | Present results of all sensitivity analyses conducted to assess the robustness of the synthesized results. | 16 |
| Reporting biases | 21 | Present assessments of risk of bias due to missing results (arising from reporting biases) for each synthesis assessed. | NA |
| Certainty of evidence | 22 | Present assessments of certainty (or confidence) in the body of evidence for each outcome assessed. | Appendix Table 34 |
| **DISCUSSION** | | |  |
| Discussion | 23a | Provide a general interpretation of the results in the context of other evidence. | 17 |
|  | 23b | Discuss any limitations of the evidence included in the review. | 20 |
|  | 23c | Discuss any limitations of the review processes used. | 20 |
|  | 23d | Discuss implications of the results for practice, policy, and future research. | 21 |
| **OTHER INFORMATION** | | |  |
| Registration and protocol | 24a | Provide registration information for the review, including register name and registration number, or state that the review was not registered. | 2 |
|  | 24b | Indicate where the review protocol can be accessed, or state that a protocol was not prepared. | 2 |
|  | 24c | Describe and explain any amendments to information provided at registration or in the protocol. | 2 |
| Support | 25 | Describe sources of financial or non-financial support for the review, and the role of the funders or sponsors in the review. | 2 |
| Competing interests | 26 | Declare any competing interests of review authors. | 2 |
| Availability of data, code and other materials | 27 | Report which of the following are publicly available and where they can be found: template data collection forms; data extracted from included studies; data used for all analyses; analytic code; any other materials used in the review. | 25 |

| **Appendix Table 2.** Search strategy | |
| --- | --- |
| Pubmed | ("pancreatitis"(MeSH Terms) OR "pancreatitis"(All Fields) OR ("acute"(All Fields) AND "pancreatitis"(All Fields)) OR "acute pancreatitis"(All Fields)) AND ("saline solution"(MeSH Terms) OR ("saline"(All Fields) AND "solution"(All Fields)) OR "saline solution"(All Fields) OR ("normal"(All Fields) AND "saline"(All Fields)) OR "normal saline"(All Fields) OR ("ringer s lactate"(MeSH Terms) OR ("ringer s"(All Fields) AND "lactate"(All Fields)) OR "ringer s lactate"(All Fields) OR ("ringer s"(All Fields) AND "lactate"(All Fields) AND "solution"(All Fields)) OR "ringer s lactate solution"(All Fields)) OR ("ringer s lactate"(MeSH Terms) OR ("ringer s"(All Fields) AND "lactate"(All Fields)) OR "ringer s lactate"(All Fields) OR ("lactated"(All Fields) AND "ringer s"(All Fields) AND "solution"(All Fields)) OR "lactated ringer s solution"(All Fields))) AND "Randomized Controlled Trial"(Publication Type) |
| Embase | ('acute pancreatitis'/exp OR 'acute pancreatitis' OR 'pancreatitis acuta') AND ('ringer lactate solution'/exp OR 'sodium chloride'/exp) AND  ('randomized controlled trial'/de OR ('randomi?ed controlled' NEXT/1 trial*) OR rct OR 'randomly allocated' OR 'allocated randomly' OR 'random allocation' OR (allocated NEAR/2 random)) |
| Cochrane | #1: (acute pancreatitis):ti,ab,kw  #2: MeSH descriptor: (Pancreatitis) explode all trees  #3: #1 or #2  #4: MeSH descriptor: (Saline Solution) explode all trees  #5: (normal saline solution):ti,ab,kw  #6: MeSH descriptor: (Ringer's Lactate) explode all trees  #7: Lactated Ringer's solution  #8: Ringer's Lactate solution  #9: #4 or #5 or #6 or #7 or #8  #10: #3 and #9 |

| **Appendix Table 3.** Reasons for exclusion of records after full-text review | | |
| --- | --- | --- |
| Study title | Citation | Excluded reason |
| A Prospective Trial of Aggressive Hydration Strategy to Reduce Post-ERCP Pancreatitis | *CENTRAL*  2020;Issue 01. | 1 |
| Aggressive intravenous hydration protocol of Lactated Ringer's solution benefits patients with mild acute pancreatitis: A meta-analysis of 5 randomized controlled trials | *Front Med (Lausanne).* 2022;9:966824. | 1 |
| Aggressive Versus Non-aggressive Goal-directed Fluid Resuscitation in Acute Pancreatitis | *CENTRAL*  2020;Issue 05. | 1 |
| Comparison of Acetate Ringer's Solution Versus Ringer's Lactated Solution for Fluid Resuscitation in Patients with Acute Pancreatitis | *CENTRAL*  2019;Issue 3. | 1 |
| Comprehensive meta-analysis of randomized controlled trials of Lactated Ringer's versus Normal Saline for acute pancreatitis | *Pancreatology*. 2021;21(8):1405-1410. | 1 |
| Does choice of intravenous fluids in acute pancreatitis really matter? An outcomes-based retrospective analysis of 20,000 military veterans | *Gastroenterology*.  2022;162(7):S98-S99. | 1 |
| Does the type of fluid used in resuscitation matter in the clinical course of acute pancreatitis? | *Indian journal of gastroenterology*.   2013; 32(SUPPL1):A110. | 1 |
| Early Fluid Resuscitation in Acute Pancreatitis: A Lot More Than Just Fluids | *Clin Gastroenterol Hepatol.*  2011;9(8):633-4. | 1 |
| Early treatment of acute pancreatitis: Do not forget the need for water | *JOP*.  2011;12(5):495-6. | 1 |
| Evaluation & safety of lactated ringer’s solution on prevention of acute pancreatitis | *CENTRAL*  2019;Issue 3. | 1 |
| Fluid therapy in acute pancreatitis: Anybody's guess | *Ann Surg.* 2013;257(2):182-8. | 1 |
| High Volume Lactated Ringer's Solution and Pancreatitis | *CENTRAL*  2018;Issue 5. | 1 |
| Intravenous fluid resuscitation in the management of acute pancreatitis | *Curr Opin Gastroenterol.*  2020;36(5):409-416. | 1 |
| Lactate Ringer's Versus Normal Saline in the Management of Acute Pancreatitis: A Systematic Review and Meta-Analysis of Randomized Controlled Trials | *Dig Dis Sci.*  2022;67(8):4131-4139. | 1 |
| Lactated Ringer Solution Is Superior to Normal Saline Solution in Managing Acute Pancreatitis: An Updated Meta-analysis of Randomized Controlled Trials | *J Clin Gastroenterol.*  2022;56(2):e114-e120. | 1 |
| Lactated Ringer's Decreases Moderately Severe/Severe Pancreatitis: Comprehensive Meta-Analysis Of Randomized Controlled Trials | *Gastroenterology.*  2021;160(6): S-295. | 1 |
| Lactated Ringers Does Not Reduce SIRS in Acute Pancreatitis Compared to Normal Saline: An Updated Meta-Analysis. | *Dig Dis Sci.*  2022;67(7):3265-3274. | 1 |
| Lactated ringer's solution does not reduce inflammation in acute pancreatitis-a meta-analysis | *Pancreatology*.  2021;21(S1):S24. | 1 |
| Lactated Ringer's vs normal saline for acute pancreatitis: An updated systematic review and meta-analysis | *Pancreatology.*  2021;21(7):1217-1223. | 1 |
| Normal Saline Versus Lactated Ringer's Solution for Fluid Resuscitation in Patients with Acute Pancreatitis: A Meta-Analysis | *Gastroenterology*.  154(6):S712. | 1 |
| Ringer's lactate versus normal saline in acute pancreatitis: A systematic review and meta-analysis | *J Dig Dis*.  2018;19(6):335-341. | 1 |
| Saline solution versus Ringer Lactate solution in the acute pancreatitis | *CENTRAL*  2019;Issue 3. | 1 |
| Specific crystalloid solutions versus normal saline for acute pancreatitis (AP) – meta-analysis with trial sequential analysis | *Pancreatology*.  2019;19(S1):S42. | 1 |
| Systematic review and meta-analysis of fluid therapy protocols in acute pancreatitis: type, rate and route | *HPB*.  2021;23(11):1629-1638. | 1 |
| The effect of aggressive Hydration in prevention of pancreatitis | *CENTRAL*  2019;Issue 3. | 1 |
| Use of Lactated Ringers Solution Compared With Normal Saline Is Associated With Shorter Length of Stay in Pediatric Acute Pancreatitis | *Pancreas*.  2020;49(3):375-380. | 2 |
| 835 Lactated ringer’s solution versus normal saline in the management of acute pancreatitis: A double-blind randomized controlled trial | *Gastroenterology*.  2020;158(6):S166. | 3 |
| Comparison of normal saline and plasmalyte in the management of acute pancreatitis | *Am J Gastroenterol.*  2019;114():p S2. | 3 |
| Comparison of normal saline versus lactated Ringer's solution for fluid resuscitation in patients with acute pancreatitis, a randomized controlled trial | *Pancreatology*.  2018;18(5):507-512. | 3 |
| Early resuscitation with lactated ringer's reduces systemic inflammation in acute pancreatitis: A multi-center randomized-controlled trial 2010 ACG governors award recipient for excellence in clinical research | *Am J Gastroenterol.*  2010;105:S70. | 3 |
| Effects of different resuscitation fluid on severe acute pancreatitis | *Gastroenterol.* 2013;19(13):2044-52. | 3 |
| In response to fluid resuscitation with lactated Ringer's solution vs. normal saline in acute pancreatitis: A triple-blind, randomized, controlled trial | *United European Gastroenterol J*. 2018;6(3):480-481. | 3 |
| Fluid resuscitation with lactated Ringer? solution vs normal saline in acute pancreatitis: A triple-blind, randomized, controlled trial | *United European Gastroenterol J*.  2018;6(1):63-72. | 3 |
| Fluid resuscitation with lactated ringer's versus normal saline in acute pancreatitis: A double-blind, randomised controlled trial | *Pancreatology*.  2016;16(3):S50. | 3 |
| Intravenous Ringers lactate versus normal saline for predominantly mild acute pancreatitis in a Nepalese Tertiary Hospital | *PLoS One*.  2022;17(1):e0263221. | 3 |
| Lactated Ringers vs Normal Saline Resuscitation for Mild Acute Pancreatitis: A Randomized Trial | *Gastroenterology.*  2021;160(3):955-957 | 3 |
| Lactated Ringer's solution reduces systemic inflammation compared with saline in patients with acute pancreatitis | *Clin Gastroenterol Hepatol.*  2011;9(8):710-717. | 3 |
| 1 = Not RCTs; 2 = Not focusing on adults with acute pancreatitis; 3 = Not focusing on aggressive vs. non-aggressive hydration protocols; 4 = No mortality outcome (primary outcome of study interest) | | |

| **Appendix Table 4.** Other characteristics of included studies | | | | | | | | | | | |
| --- | --- | --- | --- | --- | --- | --- | --- | --- | --- | --- | --- |
| First author  (year) | Type of fluids | SIRS on admission, n (%) | | BUN, mean±SD (mg/dl) | | Etiology, n (%) | | | | | |
|  |  |  |  |  |  | alcohol | | gallstone | | hyperlipidemia | |
|  |  | aggressive | non-aggressive | aggressive | non-aggressive | aggressive | non-aggressive | aggressive | non-aggressive | aggressive | non-aggressive |
| de‑Madaria  (2022) | Lactated Ringer's solution | 35 (28.7) | 29 (22.8) | 32±N/A | 36±N/A | N/A | N/A | 80 (65.6) | 71 (55.9) | N/A | N/A |
| Angsubhakorn  (2021) | Lactated Ringer's solution | 3 (13.6) | 8 (36.3) | 12.0±4.6 | 12.5±4.4 | 9(40.91) | 5(22.73) | 11 (50.0) | 10 (45.5) | 1 (4.6) | 2 (9.1) |
| Liu  (2021) | Lactated Ringer's solution | 15 (21.7) | 9 (18.8) | 11.1±3.8 | 11.0±4.6 | N/A | N/A | 6 (12.5) | 9 (13.0) | 17 (35.4)) | 29 (42.0) |
| Wang  (2020) | Lactated Ringer's solution | N/A | N/A | 13.4±2.5 | 13.6±2.6 | 10(19.2) | 9(17.3) | 15 (28.9) | 17 (32.7) | 16 (30.8) | 15 (28.9) |
| Cuéllar  (2020) | Hartmann's solution^a^ | 18 (40) | 17 (39.5) | 12.3±6.5 | 11.6±5.9 | N/A | N/A | 33 (76.7) | 37 (82.2) | N/A | N/A |
| Li  (2019) | Lactated Ringer's solution | N/A | N/A | 15.1±5.7 | 16.4±4.9 | 7(14) | 8(16) | 24 (48.0) | 26 (52.0) | 13 (26.0) | 11 (22.0) |
| Buxbaum  (2017) | Lactated Ringer's solution | 0 | 0 | 13.6±4.4 | 14.7±6.2 | 10(37) | 13(39) | N/A | N/A | N/A | N/A |
| MAO  (2010) | Crystalloid: colloid=2:1^b^ | N/A | N/A | N/A | N/A | 1(1.8) | 2(3.4) | 37 (66.1) | 45 (76.3) | 18 (32.1) | 12 (20.3) |
| MAO  (2009) | Crystalloid: colloid=2:1^b^ | N/A | N/A | N/A | N/A | N/A | N/A | N/A | N/A | N/A | N/A |
| \| ^a^ Hartmann's solution is considered similar to Lactated Ringer's solution.  ^b^ The crystalloid fluid used was either Lactated Ringer’s solution or normal saline solution; the colloid fluid was either plasma or 6% hydroxyethyl starch. \| \| --- \| | | | | | | | | | | | |

| **Appendix Table 4.** Other characteristics of included studies (continued) | |
| --- | --- |
| First author (year) | Definitions of fluid-related complications |
| de‑Madaria (2022) | This study reported a total of 33 events of fluid-related complications which were defined by at least two of the following criteria: (1) symptoms (e.g., dyspnea); (2) physical signs (e.g., peripheral edema, pulmonary rales, increased jugular venous pressure, hepatojugular reflux, or both); or (3) imaging evidence of hypervolemia. |
| Angsubhakorn (2021) | This study reported 0 event of fluid-related complications, and authors did not mention the definitions of fluid-related complications. |
| Liu (2021) | Did not evaluate the fluid-related complications. |
| Wang (2020) | Did not evaluate the fluid-related complications. |
| Cuéllar (2020) | This study reported 0 event of fluid-related complications, and authors did not mention the definitions of fluid-related complications. |
| Li (2019) | Did not evaluate the fluid-related complications. |
| Buxbaum (2017) | This study reported 0 event of fluid-related complications, defined as incident peripheral edema, pulmonary rales and ascites. |
| MAO (2010) | Did not evaluate the fluid-related complications. |
| MAO (2009) | This study reported a total of 39 events of abdominal compartment syndrome. |

| **Appendix Table 5.** Summary of risk of bias assessment among the included studies | | | | | | |
| --- | --- | --- | --- | --- | --- | --- |
|  | Bias arising from the randomization process | Bias due to deviations from intended interventions | Bias due to missing outcome data | Bias in measurement of the outcome | Bias in selection of the reported result | Overall bias* |
| Mao 2009 | Some concerns | Low | Low | Low | Some concerns | High |
| Mao 2010 | High^a^ | High^b^ | High^c^ | Low | Some concerns | High |
| Buxbaum 2017 | Low | Low | Low | Low | Some concerns | Some concerns |
| Cuéllar 2020 | Some concerns | Low | Low | Low | High^d^ | High |
| Angsubhakorn 2021 | Some concerns | Low | Low | Low | Some concerns | High |
| de-Madaria 2021 | Low | Low | Low | Low | Low | Low |
| Wang 2020 | Some concerns | Low | Low | Low | Some concerns | High |
| Li 2019 | Some concerns | Low | Low | Low | Some concerns | High |
| Liu 2021 | Some concerns | Low | Low | Low | High^d^ | High |
| *The overall bias is judged to be at low risk of bias for all domains for this result. The overall bias is judged to raise some concerns in at least one domain for this result, but not to be at high risk of bias for any domain. The overall bias is judged to be at high risk of bias in at least one domain for this result or to have some concerns for multiple domains in a way that substantially lowers confidence in the result.   1. Unknown allocated sequence concealment, but imbalanced patient characteristics 2. Unknown number of trial participants for data analysis in some outcomes 3. Unknown number of trial participants for data analysis in some outcomes, and data absence may have depended on true value 4. Results selected from multiple outcomes during different time points | | | | | | |

| **Appendix Table 6.** Subgroup analyses: Mortality | | | | |
| --- | --- | --- | --- | --- |
|  | **Number of studies** | **Number of participants** | **Relative risk (95% CI)** | **I^2^ value** |
| 1. Countries |  |  |  |  |
| Severe acute pancreatitis |  |  |  |  |
| Asian | 2 | 191 | 2.45 (1.37, 4.40) | 0% |
| Non-Asian | - | - | - | - |
| Non-severe acute pancreatitis |  |  |  |  |
| Asian | 4 | 365 | 2.88 (0.27, 30.82) | Not applicable |
| Non-Asian | 3 | 397 | 1.74 (0.19, 15.88) | 29% |
| 2. Mean age |  |  |  |  |
| Severe acute pancreatitis |  |  |  |  |
| <50 years old | 1 | 115 | 2.22 (1.10, 4.50) | Not applicable |
| <50 years old | 1 | 76 | 3.06 (1.07, 8.75) | Not applicable |
| Non-severe acute pancreatitis |  |  |  |  |
| <50 years old | 6 | 513 | 1.42 (0.21, 9.46) | 0% |
| <50 years old | 1 | 249 | 4.16 (0.47, 36.73) | Not applicable |

| **Appendix Table 7.** Subgroup analyses: Clinical improvement | | | | |
| --- | --- | --- | --- | --- |
|  | **Number of studies** | **Number of participants** | **Relative risk (95% CI)** | **I^2^ value** |
| 1. Countries |  |  |  |  |
| Severe acute pancreatitis |  |  |  |  |
| Asian | - | - | - | - |
| Non-Asian | - | - | - | - |
| Non-severe acute pancreatitis |  |  |  |  |
| Asian | 1 | 44 | 0.86 (0.52, 1.41) | Not applicable |
| Non-Asian | 1 | 60 | 1.66 (1.04, 2.65) | Not applicable |
| 2. Mean age |  |  |  |  |
| Severe acute pancreatitis |  |  |  |  |
| <50 years old | 2 | 104 | 1.20 (0.63, 2.29) | 72% |
| >50 years old | - | - | - | - |
| Non-severe acute pancreatitis |  |  |  |  |
| <50 years old | - | - | - | - |
| >50 years old | - | - | - | - |

| **Appendix Table 8.** Subgroup analyses: APACHE II score changes | | | | |
| --- | --- | --- | --- | --- |
|  | **Number of studies** | **Number of participants** | **Mean difference (95% CI)** | **I^2^ value** |
| 1. Countries |  |  |  |  |
| Severe acute pancreatitis |  |  |  |  |
| Asian | 2 | 191 | 3.31 (1.79, 4.84) | 0% |
| Non-Asian | - | - | - | - |
| Non-severe acute pancreatitis |  |  |  |  |
| Asian | - | - | - | - |
| Non-Asian | - | - | - | - |
| 2. Mean age |  |  |  |  |
| Severe acute pancreatitis |  |  |  |  |
| <50 years old | 1 | 115 | 3.10 (2.40, 3.80) | Not applicable |
| >50 years old | 1 | 76 | 3.60 (2.80, 4.40) | Not applicable |
| Non-severe acute pancreatitis |  |  |  |  |
| <50 years old | - | - | - | - |
| >50 years old | - | - | - | - |

| **Appendix Table 9.** Subgroup analyses: Fluid-related complications | | | | |
| --- | --- | --- | --- | --- |
|  | **Number of studies** | **Number of participants** | **Relative risk (95% CI)** | **I^2^ value** |
| 1. Countries |  |  |  |  |
| Severe acute pancreatitis |  |  |  |  |
| Asian | 1 | 76 | 2.22 (1.36, 3.63) | Not applicable |
| Non-Asian | - | - | - | - |
| Non-severe acute pancreatitis |  |  |  |  |
| Asian | - | - | - | - |
| Non-Asian | 3 | 353 | 3.25 (1.53, 6.93) | Not applicable |
| 2. Mean age |  |  |  |  |
| Severe acute pancreatitis |  |  |  | Not applicable |
| <50 years old | - | - | - | - |
| >50 years old | 1 | 76 | 2.22 (1.36, 3.63) | Not applicable |
| Non-severe acute pancreatitis |  |  |  |  |
| <50 years old | 2 | 104 | Not estimable | Not applicable |
| >50 years old | 1 | 249 | 3.25 (1.53, 6.93) | Not applicable |

| **Appendix Table 10.** Subgroup analyses: Acute respiratory failure | | | | |
| --- | --- | --- | --- | --- |
|  | **Number of studies** | **Number of participants** | **Relative risk (95% CI)** | **I^2^ value** |
| 1. Countries |  |  |  |  |
| Severe acute pancreatitis |  |  |  |  |
| Asian | 1 | 76 | 1.45 (1.14, 1.85) | Not applicable |
| Non-Asian | - | - | - | - |
| Non-severe acute pancreatitis |  |  |  |  |
| Asian | 1 | 117 | 1.44 (0.21, 9.85) | Not applicable |
| Non-Asian | 1 | 249 | 3.12 (0.87, 11.26) | Not applicable |
| 2. Mean age |  |  |  |  |
| Severe acute pancreatitis |  |  |  |  |
| <50 years old | - | - | - | - |
| >50 years old | 1 | 76 | 1.45 (1.14, 1.85) | Not applicable |
| Non-severe acute pancreatitis |  |  |  |  |
| <50 years old | - | - | - | - |
| >50 years old | 1 | 249 | 3.12 (0.87, 11.26) | Not applicable |

| **Appendix Table 11.** Subgroup analyses: Sepsis | | | | |
| --- | --- | --- | --- | --- |
|  | **Number of studies** | **Number of participants** | **Relative risk (95% CI)** | **I^2^ value** |
| 1. Countries |  |  |  |  |
| Severe acute pancreatitis |  |  |  |  |
| Asian | 2 | 191 | 1.44 (1.15, 1.80) | 0% |
| Non-Asian | - | - | - | - |
| Non-severe acute pancreatitis |  |  |  |  |
| Asian | - | - | - | - |
| Non-Asian | 1 | 249 | 1.73 (0.42, 7.10) | Not applicable |
| 2. Mean age |  |  |  |  |
| Severe acute pancreatitis |  |  |  |  |
| <50 years old | 1 | 115 | 1.36 (1.05, 1.76) | Not applicable |
| >50 years old | 1 | 76 | 1.70 (1.07, 2.72) | Not applicable |
| Non-severe acute pancreatitis |  |  |  |  |
| <50 years old | - | - | - | - |
| >50 years old | 1 | 249 | 1.73 (0.42, 7.10) | Not applicable |

| **Appendix Table 12.** Subgroup analyses: Acute kidney injury | | | | |
| --- | --- | --- | --- | --- |
|  | **Number of studies** | **Number of participants** | **Relative risk (95% CI)** | **I^2^ value** |
| 1. Countries |  |  |  |  |
| Severe acute pancreatitis |  |  |  |  |
| Asian | - | - | - | - |
| Non-Asian | - | - | - | - |
| Non-severe acute pancreatitis |  |  |  |  |
| Asian | 1 | 104 | 0.33 (0.01, 8.00) | Not applicable |
| Non-Asian | 2 | 337 | 0.91 (0.33, 2.47) | 0% |
| 2. Mean age |  |  |  |  |
| Severe acute pancreatitis |  |  |  |  |
| <50 years old | - | - | - | - |
| >50 years old | - | - | - | - |
| Non-severe acute pancreatitis |  |  |  |  |
| <50 years old | 2 | 192 | 0.57 (0.16, 2.00) | 0% |
| >50 years old | 1 | 249 | 1.39 (0.32, 6.07) | Not applicable |

| **Appendix Table 13.** Subgroup analyses: Pancreatic necrosis | | | | |
| --- | --- | --- | --- | --- |
|  | **Number of studies** | **Number of participants** | **Relative risk (95% CI)** | **I^2^ value** |
| 1. Countries |  |  |  |  |
| Severe acute pancreatitis |  |  |  |  |
| Asian | - | - | - | - |
| Non-Asian | - | - | - | - |
| Non-severe acute pancreatitis |  |  |  |  |
| Asian | - | - | - | - |
| Non-Asian | 2 | 337 | 1.82 (0.92, 3.59) | 0% |
| 2. Mean age |  |  |  |  |
| Severe acute pancreatitis |  |  |  |  |
| <50 years old | - | - | - | - |
| >50 years old | - | - | - | - |
| Non-severe acute pancreatitis |  |  |  |  |
| <50 years old | 1 | 88 | 1.40 (0.33, 5.87) | Not applicable |
| >50 years old | 1 | 249 | 1.97 (0.91, 4.24) | Not applicable |

| **Appendix Table 14.** Subgroup analyses: SIRS subsiding within 48 hours | | | | |
| --- | --- | --- | --- | --- |
|  | **Number of studies** | **Number of participants** | **Relative risk (95% CI)** | **I^2^ value** |
| 1. Countries |  |  |  |  |
| Severe acute pancreatitis |  |  |  |  |
| Asian | - | - | - | - |
| Non-Asian | - | - | - | - |
| Non-severe acute pancreatitis |  |  |  |  |
| Asian | 1 | 44 | 0.40 (0.09, 1.85) | Not applicable |
| Non-Asian | 3 | 397 | 1.20 (0.75, 1.91) | 0% |
| 2. Mean age |  |  |  |  |
| Severe acute pancreatitis |  |  |  |  |
| <50 years old | - | - | - | - |
| >50 years old | - | - | - | - |
| Non-severe acute pancreatitis |  |  |  |  |
| <50 years old | 3 | 192 | 0.92 (0.51, 1.66) | 0% |
| >50 years old | 1 | 249 | 1.36 (0.69, 2.68) | Not applicable |

| **Appendix Table 15.** Subgroup analyses: SIRS persisting >48 hours | | | | |
| --- | --- | --- | --- | --- |
|  | **Number of studies** | **Number of participants** | **Relative risk (95% CI)** | **I^2^ value** |
| 1. Countries |  |  |  |  |
| Severe acute pancreatitis |  |  |  |  |
| Asian | - | - | - | - |
| Non-Asian | - | - | - | - |
| Non-severe acute pancreatitis |  |  |  |  |
| Asian | - | - | - | - |
| Non-Asian | 3 | 348 | 1.04 (0.50, 2.16) | 30% |
| 2. Mean age |  |  |  |  |
| Severe acute pancreatitis |  |  |  |  |
| <50 years old | - | - | - | - |
| >50 years old | - | - | - | - |
| Non-severe acute pancreatitis |  |  |  |  |
| <50 years old | 2 | 148 | 0.74 (0.23, 2.43) | 48% |
| >50 years old | 1 | 200 | 1.55 (0.61, 3.90) | Not applicable |

| **Appendix Table 16.** Subgroup analyses: Persistent organ failure | | | | |
| --- | --- | --- | --- | --- |
|  | **Number of studies** | **Number of participants** | **Relative risk (95% CI)** | **I^2^ value** |
| 1. Countries |  |  |  |  |
| Severe acute pancreatitis |  |  |  |  |
| Asian | - | - | - | - |
| Non-Asian | - | - | - | - |
| Non-severe acute pancreatitis |  |  |  |  |
| Asian | 2 | 161 | 0.72 (0.23, 2.25) | Not applicable |
| Non-Asian | 3 | 397 | 1.73 (0.76, 3.98) | 13% |
| 2. Mean age |  |  |  |  |
| Severe acute pancreatitis |  |  |  |  |
| <50 years old | - | - | - | - |
| >50 years old | - | - | - | - |
| Non-severe acute pancreatitis |  |  |  |  |
| <50 years old | 4 | 309 | 1.09 (0.57, 2.09) | 0% |
| >50 years old | 1 | 249 | 4.16 (0.90, 19.22) | Not applicable |

| **Appendix Table 17.** Subgroup analyses: BUN changes within 48 hours. | | | | |
| --- | --- | --- | --- | --- |
|  | **Number of studies** | **Number of participants** | **Mean difference (95% CI)** | **I^2^ value** |
| 1. Countries |  |  |  |  |
| Severe acute pancreatitis |  |  |  |  |
| Asian | - | - | - | - |
| Non-Asian | - | - | - | - |
| Non-severe acute pancreatitis |  |  |  |  |
| Asian | 1 | 44 | -2.12 (-4.93, 0.69) | Not applicable |
| Non-Asian | 1 | 60 | -1.60 (-4.23, 1.03) | Not applicable |
| 2. Mean age |  |  |  |  |
| Severe acute pancreatitis |  |  |  |  |
| <50 years old | - | - | - | - |
| >50 years old | - | - | - | - |
| Non-severe acute pancreatitis |  |  |  |  |
| <50 years old | 2 | 104 | -1.84 (-3.76, 0.08) | 0% |
| >50 years old | - | - | - | - |

| **Appendix Table 18.** Subgroup analyses: Hct changes within 48 hours | | | | |
| --- | --- | --- | --- | --- |
|  | **Number of studies** | **Number of participants** | **Mean difference (95% CI)** | **I^2^ value** |
| 1. Countries |  |  |  |  |
| Severe acute pancreatitis |  |  |  |  |
| Asian | 2 | 191 | -4.41 (-15.97, 7.16] | 100% |
| Non-Asian | - | - | - | - |
| Non-severe acute pancreatitis |  |  |  |  |
| Asian | 1 | 44 | 0.11 (-1.93, 2.15) | Not applicable |
| Non-Asian | 1 | 60 | -2.60 (-4.63, -0.57) | Not applicable |
| 2. Mean age |  |  |  |  |
| Severe acute pancreatitis |  |  |  |  |
| <50 years old | 1 | 114 | -10.30 (-10.81, -9.79] | Not applicable |
| >50 years old | 1 | 76 | 1.50 (0.63, 2.37] | Not applicable |
| Non-severe acute pancreatitis |  |  |  | Not applicable |
| <50 years old | 2 | 104 | -1.25 (-3.90, 1.41) | 71% |
| >50 years old | - | - | - | - |

| **Appendix Table 19.** Subgroup analyses: Total hospitalization days | | | | |
| --- | --- | --- | --- | --- |
|  | **Number of studies** | **Number of participants** | **Mean difference (95% CI)** | **I^2^ value** |
| 1. Countries |  |  |  |  |
| Severe acute pancreatitis |  |  |  |  |
| Asian | - | - | - | - |
| Non-Asian | - | - | - | - |
| Non-severe acute pancreatitis |  |  |  |  |
| Asian | 4 | 364 | -0.93 (-3.39, 1.53) | 98% |
| Non-Asian | 2 | 309 | 0.53 (-0.45, 1.51) | 92% |
| 2. Mean age |  |  |  |  |
| Severe acute pancreatitis |  |  |  |  |
| <50 years old | - | - | - | - |
| >50 years old | - | - | - | - |
| Non-severe acute pancreatitis |  |  |  |  |
| <50 years old | 5 | 424 | -0.73 (-2.56, 1.09) | 97% |
| >50 years old | 1 | 249 | 1.00 (0.75, 1.25) | Not applicable |

| **Appendix Table 20.** Subgroup analyses of sensitivity analyses: Mortality | | | | |
| --- | --- | --- | --- | --- |
|  | **Number of studies** | **Number of participants** | **Relative risk (95% CI)** | **I^2^ value** |
| 1. Countries |  |  |  |  |
| Severe acute pancreatitis |  |  |  |  |
| Asian | 2 | 191 | 2.45 (1.37, 4.40) | 0% |
| Non-Asian | - | - | - | - |
| Non-severe acute pancreatitis |  |  |  |  |
| Asian | 3 | 261 | 2.88 (0.27, 30.82) | Not applicable |
| Non-Asian | 2 | 309 | 1.74 (0.19, 15.88) | 29% |
| 2. Mean age |  |  |  |  |
| Severe acute pancreatitis |  |  |  |  |
| <50 years old | 1 | 115 | 2.22 (1.10, 4.50) | Not applicable |
| >50 years old | 1 | 76 | 3.06 (1.07, 8.75) | Not applicable |
| Non-severe acute pancreatitis |  |  |  |  |
| <50 years old | 4 | 321 | 1.42 (0.21, 9.46) | 0% |
| >50 years old | 1 | 249 | 4.16 (0.47, 36.73) | Not applicable |

| **Appendix Table 21.** Subgroup analyses of sensitivity analyses: Clinical improvement | | | | |
| --- | --- | --- | --- | --- |
|  | **Number of studies** | **Number of participants** | **Relative risk (95% CI)** | **I^2^ value** |
| 1. Countries |  |  |  |  |
| Severe acute pancreatitis |  |  |  |  |
| Asian | - | - | - | - |
| Non-Asian | - | - | - | - |
| Non-severe acute pancreatitis |  |  |  |  |
| Asian | 1 | 44 | 0.86 (0.52, 1.41) | Not applicable |
| Non-Asian | 1 | 60 | 1.66 (1.04, 2.65) | Not applicable |
| 2. Mean age |  |  |  |  |
| Severe acute pancreatitis |  |  |  |  |
| <50 years old | - | - | - | - |
| >50 years old | - | - | - | - |
| Non-severe acute pancreatitis |  |  |  |  |
| <50 years old | 2 | 104 | 1.20 (0.63, 2.29) | 72% |
| >50 years old | - | - | - | - |

| **Appendix Table 22.** Subgroup analyses: APACHE II score changes | | | | | |
| --- | --- | --- | --- | --- | --- |
|  | **Number of studies** | **Number of participants** | **Mean difference (95% CI)** | **I^2^ value** |  |
| 1. Countries |  |  |  |  |  |
| Severe acute pancreatitis |  |  |  |  |  |
| Asian | 2 | 191 | 3.31 (1.79, 4.84) | 0% |  |
| Non-Asian | - | - | - | - |  |
| Non-severe acute pancreatitis |  |  |  |  |  |
| Asian | - | - | - | - |  |
| Non-Asian | - | - | - | - |  |
| 2. Mean age |  |  |  |  |  |
| Severe acute pancreatitis |  |  |  |  |  |
| <50 years old | 1 | 115 | 3.10 (2.40, 3.80) | Not applicable |  |
| >50 years old | 1 | 76 | 3.60 (2.80, 4.40) | Not applicable |  |
| Non-severe acute pancreatitis |  |  |  |  |  |
| <50 years old | - | - | - | - |  |
| >50 years old | - | - | - | - |  |

| **Appendix Table 23.** Subgroup analyses of sensitivity analyses: Fluid-related complications | | | | |
| --- | --- | --- | --- | --- |
|  | Number of studies | Number of participants | Relative risk (95% CI) | I^2^ value |
| 1. Countries |  |  |  |  |
| Severe acute pancreatitis |  |  |  |  |
| Asian | 1 | 76 | 2.22 (1.36, 3.63) | Not applicable |
| Non-Asian | - | - | - | - |
| Non-severe acute pancreatitis |  |  |  |  |
| Asian | 1 | 44 | Not estimable | Not applicable |
| Non-Asian | 2 | 309 | 3.25 (1.53, 6.93) | Not applicable |
| 2. Mean age |  |  |  |  |
| Severe acute pancreatitis |  |  |  |  |
| <50 years old | - | - | - | - |
| >50 years old | 1 | 76 | 2.22 (1.36, 3.63) | Not applicable |
| Non-severe acute pancreatitis |  |  |  | Not applicable |
| <50 years old | 2 | 104 | Not estimable | Not applicable |
| >50 years old | 1 | 249 | 3.25 (1.53, 6.93) | Not applicable |

| **Appendix Table 24.** Subgroup analyses of sensitivity analyses: Acute respiratory failure | | | | |
| --- | --- | --- | --- | --- |
|  | **Number of studies** | **Number of participants** | **Relative risk (95% CI)** | **I^2^ value** |
| 1. Countries |  |  |  |  |
| Severe acute pancreatitis |  |  |  |  |
| Asian | 1 | 76 | 1.45 (1.14, 1.85) | Not applicable |
| Non-Asian | - | - | - | - |
| Non-severe acute pancreatitis |  |  |  |  |
| Asian | - | - | - | - |
| Non-Asian | 1 | 249 | 3.12 (0.87, 11.26) | Not applicable |
| 2. Mean age |  |  |  |  |
| Severe acute pancreatitis |  |  |  |  |
| <50 years old | - | - | - | - |
| >50 years old | 1 | 76 | 1.45 (1.14, 1.85) | Not applicable |
| Non-severe acute pancreatitis |  |  |  |  |
| <50 years old | - | - | - | - |
| >50 years old | 1 | 249 | 3.12 (0.87, 11.26) | Not applicable |

| **Appendix Table 25.** Subgroup analyses of sensitivity analyses: Sepsis | | | | |
| --- | --- | --- | --- | --- |
|  | **Number of studies** | **Number of participants** | **Relative risk (95% CI)** | **I^2^ value** |
| 1. Countries |  |  |  |  |
| Severe acute pancreatitis |  |  |  |  |
| Asian | 2 | 191 | 1.44 (1.15, 1.80) | 0% |
| Non-Asian | - | - | - | - |
| Non-severe acute pancreatitis |  |  |  |  |
| Asian | - | - | - | - |
| Non-Asian | 1 | 249 | 1.73 (0.42, 7.10) | Not applicable |
| 2. Mean age |  |  |  |  |
| Severe acute pancreatitis |  |  |  |  |
| <50 years old | 1 | 115 | 1.36 (1.05, 1.76) | Not applicable |
| >50 years old | 1 | 76 | 1.70 (1.07, 2.72) | Not applicable |
| Non-severe acute pancreatitis |  |  |  |  |
| <50 years old | - | - | - | - |
| >50 years old | 1 | 249 | 1.73 (0.42, 7.10) | Not applicable |

| **Appendix Table 26.** Subgroup analyses of sensitivity analyses: Acute kidney injury | | | | |
| --- | --- | --- | --- | --- |
|  | **Number of studies** | **Number of participants** | **Relative risk (95% CI)** | **I^2^ value** |
| 1. Countries |  |  |  |  |
| Severe acute pancreatitis |  |  |  |  |
| Asian | - | - | - | - |
| Non-Asian | - | - | - | - |
| Non-severe acute pancreatitis |  |  |  |  |
| Asian | - | - | - | - |
| Non-Asian | 1 | 249 | 1.11 (0.29, 4.24) | Not applicable |
| 2. Mean age |  |  |  |  |
| Severe acute pancreatitis |  |  |  |  |
| <50 years old | - | - | - | - |
| >50 years old | - | - | - | - |
| Non-severe acute pancreatitis |  |  |  |  |
| <50 years old | - | - | - | - |
| >50 years old | 1 | 249 | 1.39 (0.32, 6.07) | Not applicable |

| **Appendix Table 27.** Subgroup analyses of sensitivity analyses: Pancreatic necrosis | | | | |
| --- | --- | --- | --- | --- |
|  | **Number of studies** | **Number of participants** | **Relative risk (95% CI)** | **I^2^ value** |
| 1. Countries |  |  |  |  |
| Severe acute pancreatitis |  |  |  |  |
| Asian | - | - | - | - |
| Non-Asian | - | - | - | - |
| Non-severe acute pancreatitis |  |  |  |  |
| Asian | - | - | - | - |
| Non-Asian | 1 | 249 | 1.97 (0.91, 4.24) | Not applicable |
| 2. Mean age |  |  |  |  |
| Severe acute pancreatitis |  |  |  |  |
| <50 years old | - | - | - | - |
| >50 years old | - | - | - | - |
| Non-severe acute pancreatitis |  |  |  |  |
| <50 years old | - | - | - | - |
| >50 years old | 1 | 249 | 1.97 (0.91, 4.24) | Not applicable |

| **Appendix Table 28.** Subgroup analyses of sensitivity analyses: SIRS subsiding within 48 hours | | | | |
| --- | --- | --- | --- | --- |
|  | **Number of studies** | **Number of participants** | **Relative risk (95% CI)** | **I^2^ value** |
| 1. Countries |  |  |  |  |
| Severe acute pancreatitis |  |  |  |  |
| Asian | - | - | - | - |
| Non-Asian | - | - | - | - |
| Non-severe acute pancreatitis |  |  |  |  |
| Asian | 1 | 44 | 0.40 (0.09, 1.85) | Not applicable |
| Non-Asian | 2 | 309 | 1.34 (0.71, 2.55) | 0% |
| 2. Mean age |  |  |  |  |
| Severe acute pancreatitis |  |  |  |  |
| <50 years old | - | - | - | - |
| >50 years old | - | - | - | - |
| Non-severe acute pancreatitis |  |  |  |  |
| <50 years old | 2 | 104 | 0.62 (0.19, 2.04) | 0% |
| >50 years old | 1 | 249 | 1.36 (0.69, 2.68) | Not applicable |

| **Appendix Table 29.** Subgroup analyses of sensitivity analyses: SIRS persisting >48 hours | | | | |
| --- | --- | --- | --- | --- |
|  | **Number of studies** | **Number of participants** | **Relative risk (95% CI)** | **I^2^ value** |
| 1. Countries |  |  |  |  |
| Severe acute pancreatitis |  |  |  |  |
| Asian | - | - | - | - |
| Non-Asian | - | - | - | - |
| Non-severe acute pancreatitis |  |  |  |  |
| Asian | - | - | - | - |
| Non-Asian | 2 | 260 | 0.83 (0.19, 3.51) | 64% |
| 2. Mean age |  |  |  |  |
| Severe acute pancreatitis |  |  |  |  |
| <50 years old | - | - | - | - |
| >50 years old | - | - | - | - |
| Non-severe acute pancreatitis |  |  |  |  |
| <50 years old | 1 | 60 | 0.35 (0.08, 1.54) | Not applicable |
| >50 years old | 1 | 200 | 1.55 (0.61, 3.90) | Not applicable |

| **Appendix Table 30.** Subgroup analyses of sensitivity analyses: Persistent organ failure | | | | |
| --- | --- | --- | --- | --- |
|  | **Number of studies** | **Number of participants** | **Relative risk (95% CI)** | **I^2^ value** |
| 1. Countries |  |  |  |  |
| Severe acute pancreatitis |  |  |  |  |
| Asian | - | - | - | - |
| Non-Asian | - | - | - | - |
| Non-severe acute pancreatitis |  |  |  |  |
| Asian | 2 | 161 | 0.72 (0.23, 2.25) | Not applicable |
| Non-Asian | 2 | 309 | 1.99 (0.24, 16.68) | 41% |
| 2. Mean age |  |  |  |  |
| Severe acute pancreatitis |  |  |  |  |
| <50 years old | - | - | - | - |
| >50 years old | - | - | - | - |
| Non-severe acute pancreatitis |  |  |  |  |
| <50 years old | 3 | 221 | 0.67 (0.23, 1.97) | 0% |
| >50 years old | 1 | 249 | 4.16 (0.90, 19.22) | Not applicable |

| **Appendix Table 31.** Subgroup analyses of sensitivity analyses: BUN changes within 48 hours. | | | | |
| --- | --- | --- | --- | --- |
|  | **Number of studies** | **Number of participants** | **Mean difference (95% CI)** | **I^2^ value** |
| 1. Countries |  |  |  |  |
| Severe acute pancreatitis |  |  |  |  |
| Asian | - | - | - | - |
| Non-Asian | - | - | - | - |
| Non-severe acute pancreatitis |  |  |  |  |
| Asian | 1 | 44 | -2.12 (-4.93, 0.69) | Not applicable |
| Non-Asian | 1 | 60 | -1.60 (-4.23, 1.03) | Not applicable |
| 2. Mean age |  |  |  |  |
| Severe acute pancreatitis |  |  |  |  |
| <50 years old | - | - | - | - |
| >50 years old | - | - | - | - |
| Non-severe acute pancreatitis |  |  |  |  |
| <50 years old | 2 | 104 | -1.84 (-3.76, 0.08) | 0% |
| >50 years old | - | - | - | - |

| **Appendix Table 32.** Subgroup analyses of sensitivity analyses: Hct changes within 48 hours. | | | | |
| --- | --- | --- | --- | --- |
|  | **Number of studies** | **Number of participants** | **Mean difference (95% CI)** | **I^2^ value** |
| 1. Countries |  |  |  |  |
| Severe acute pancreatitis |  |  |  |  |
| Asian | 2 | 191 | -4.41 (-15.97, 7.16) | 100% |
| Non-Asian | - | - | - | - |
| Non-severe acute pancreatitis |  |  |  |  |
| Asian | 1 | 44 | 0.11 (-1.93, 2.15) | Not applicable |
| Non-Asian | 1 | 60 | -2.60 (-4.63, -0.57) | Not applicable |
| 2. Mean age |  |  |  |  |
| Severe acute pancreatitis |  |  |  |  |
| <50 years old | 1 | 114 | -10.30 (-10.81, -9.79) | Not applicable |
| >50 years old | 1 | 76 | 1.50 (0.63, 2.37) | Not applicable |
| Non-severe acute pancreatitis |  |  |  | Not applicable |
| <50 years old | 2 | 104 | -1.25 (-3.90, 1.41) | 71% |
| >50 years old | - | - | - | - |

| **Appendix Table 33.** Subgroup analyses of sensitivity analyses: Total hospitalization days | | | | |
| --- | --- | --- | --- | --- |
|  | **Number of studies** | **Number of participants** | **Mean difference (95% CI)** | **I^2^ value** |
| 1. Countries |  |  |  |  |
| Severe acute pancreatitis |  |  |  |  |
| Asian | - | - | - | - |
| Non-Asian | - | - | - | - |
| Non-severe acute pancreatitis |  |  |  |  |
| Asian | 3 | 260 | -0.46 (-3.51, 2.59) | 98% |
| Non-Asian | 1 | 249 | 1.00 (0.75, 1.25) | Not applicable |
| 2. Mean age |  |  |  |  |
| Severe acute pancreatitis |  |  |  |  |
| <50 years old | - | - | - | - |
| >50 years old | - | - | - | - |
| Non-severe acute pancreatitis |  |  |  |  |
| <50 years old | 3 | 260 | -0.46 (-3.51, 2.59) | 98% |
| >50 years old | 1 | 249 | 1.00 (0.75, 1.25) | Not applicable |

| **Appendix Table 34.** GRADE assessment on study outcomes of the included RCTs | | | | | | |
| --- | --- | --- | --- | --- | --- | --- |
|  | Risk of bias^a^ | Inconsistency^b^ | Indirectness | Imprecision^c^ | Publication bias^d^ | Certainty of evidence |
| **Mortality** |  |  |  |  |  |  |
| Severe acute pancreatitis | Serious | No concern | No concern | Very serious | Not available | Very Low |
| Non-severe acute pancreatitis | Serious | No concern | No concern | Very serious | Not available | Very Low |
| **Clinical improvement** |  |  |  |  |  |  |
| Non-severe acute pancreatitis | Serious | Serious | No concern | Serious | Not available | Very Low |
| **APACHE II score changes** |  |  |  |  |  |  |
| Severe acute pancreatitis | Serious | No concern | No concern | Very serious | Not available | Very Low |
| **Fluid-related complications** |  |  |  |  |  |  |
| Severe acute pancreatitis | Serious | No concern | No concern | Very serious | Not available | Very Low |
| Non-severe acute pancreatitis | Serious | No concern | No concern | Very serious | Not available | Very Low |
| **Acute respiratory failure** |  |  |  |  |  |  |
| Non-severe acute pancreatitis | Serious | No concern | No concern | Serious | Not available | Low |
| **Sepsis** |  |  |  |  |  |  |
| Severe acute pancreatitis | Serious | No concern | No concern | Serious | Not available | Low |
| Non-severe acute pancreatitis | Serious | No concern | No concern | Serious | Not available | Low |
| **SIRS subsiding** |  |  |  |  |  |  |
| Non-severe acute pancreatitis | Serious | No concern | No concern | Serious | Not available | Low |
| **Acute kidney injury** |  |  |  |  |  |  |
| Non-severe acute pancreatitis | Serious | No concern | No concern | Serious | Not available | Low |
| **Pancreatic necrosis** |  |  |  |  |  |  |
| Non-severe acute pancreatitis | Serious | No concern | No concern | Serious | Not available | Low |
| **Clinical progression by Atlanta classification** |  |  |  |  |  |  |
| Severe acute pancreatitis | Serious | No concern | No concern | Serious | Not available | Low |
| **SIRS persisting >48 hours** |  |  |  |  |  |  |
| Non-severe acute pancreatitis | Serious | No concern | No concern | Serious | Not available | Low |
| **Persistent organ failure** |  |  |  |  |  |  |
| From mild to severe acute pancreatitis | Serious | No concern | No concern | Serious | Not available | Low |
| **Hct changes within 48 hours** |  |  |  |  |  |  |
| Non-severe acute pancreatitis | Serious | Serious | No concern | Serious | Not available | Very Low |
| **BUN changes within 48 hours** |  |  |  |  |  |  |
| Non-severe acute pancreatitis | Serious | Serious | No concern | Serious | Not available | Very Low |
| **Total hospitalization days** |  |  |  |  |  |  |
| Non-severe acute pancreatitis | Serious | Serious | No concern | Serious | Not available | Very Low |
| ^a^We downgraded the certainty of evidence if the study biases substantially reduced the confidence in a point estimate.  ^b^We referred to the I^2^ value as a measure of inconsistency.  ^c^We referred to the minimal clinically important difference and confidence intervals of the absolute effect to rate imprecision.  ^d^We could not perform the analysis of publication bias due to the number of included RCTs being less than 10. | | | | | | |
